# The International SSRI Pharmacogenomics Consortium (ISPC): a genome-wide association study of antidepressant treatment response

**DOI:** 10.1038/tp.2015.47

**Published:** 2015-04-21

**Authors:** J M Biernacka, K Sangkuhl, G Jenkins, R M Whaley, P Barman, A Batzler, R B Altman, V Arolt, J Brockmöller, C H Chen, K Domschke, D K Hall-Flavin, C J Hong, A Illi, Y Ji, O Kampman, T Kinoshita, E Leinonen, Y J Liou, T Mushiroda, S Nonen, M K Skime, L Wang, B T Baune, M Kato, Y L Liu, V Praphanphoj, J C Stingl, S J Tsai, M Kubo, T E Klein, R Weinshilboum

**Affiliations:** 1 Department of Psychiatry and Psychology, Mayo Clinic, Rochester, MN, USA; 2 Department of Health Sciences Research, Mayo Clinic, Rochester, MN, USA; 3 Department of Genetics, Stanford University, Stanford, CA, USA; 4 Department of Bioengineering, Stanford University, Stanford, CA, USA; 5 Department of Psychiatry and Psychotherapy, University of Muenster, Muenster, Germany; 6 Department of Clinical Pharmacology, University Göttingen, Göttingen, Germany; 7 Department of Psychiatry, Taipei Medical University-Shuangho Hospital, New Taipei City, Taiwan; 8 Department of Psychiatry, Psychosomatics and Psychotherapy, University of Wuerzburg, Wuerzburg, Germany; 9 Department of Psychiatry, Taipei Veterans General Hospital, Taipei, Taiwan; 10 Division of Psychiatry, School of Medicine, National Yang-Ming University, Taipei, Taiwan; 11 Department of Psychiatry, School of Medicine, University of Tampere, Tampere, Finland; 12 Department of Molecular Pharmacology and Experimental Therapeutics, Mayo Clinic, Rochester, MN, USA; 13 Department of Psychiatry, Seinäjoki Hospital District, Seinäjoki, Finland; 14 Department of Neuropsychiatry, Kansai Medical University, Osaka, Japan; 15 Department of Psychiatry, Tampere University Hospital, Tampere, Finland; 16 RIKEN Center for Integrative Medical Sciences, Kanagawa, Japan; 17 Department of Pharmacy, Hyogo University of Health Sciences, Hyogo, Japan; 18 Department of Psychiatry, University of Adelaide, Adelaide, SA, Australia; 19 Center for Neuropsychiatric Research, National Health Research Institutes, Miaoli, Taiwan; 20 Center for Medical Genetics Research, Rajanukul Institute, Department of Mental Health, Ministry of Public Health Bangkok, Bangkok, Thailand; 21 Research Division Federal Institute for Drugs and Medical Devices, Bonn, Germany

## Abstract

Response to treatment with selective serotonin reuptake inhibitors (SSRIs) varies considerably between patients. The International SSRI Pharmacogenomics Consortium (ISPC) was formed with the primary goal of identifying genetic variation that may contribute to response to SSRI treatment of major depressive disorder. A genome-wide association study of 4-week treatment outcomes, measured using the 17-item Hamilton Rating Scale for Depression (HRSD-17), was performed using data from 865 subjects from seven sites. The primary outcomes were percent change in HRSD-17 score and response, defined as at least 50% reduction in HRSD-17. Data from two prior studies, the Pharmacogenomics Research Network Antidepressant Medication Pharmacogenomics Study (PGRN-AMPS) and the Sequenced Treatment Alternatives to Relieve Depression (STAR*D) study, were used for replication, and a meta-analysis of the three studies was performed (*N*=2394). Although many top association signals in the ISPC analysis map to interesting candidate genes, none were significant at the genome-wide level and the associations were not replicated using PGRN-AMPS and STAR*D data. Top association results in the meta-analysis of response included single-nucleotide polymorphisms (SNPs) in the *HPRTP4* (hypoxanthine phosphoribosyltransferase pseudogene 4)/*VSTM5* (V-set and transmembrane domain containing 5) region, which approached genome-wide significance (*P*=5.03E−08) and SNPs 5' upstream of the neuregulin-1 gene, *NRG1* (*P*=1.20E−06). *NRG1* is involved in many aspects of brain development, including neuronal maturation and variations in this gene have been shown to be associated with increased risk for mental disorders, particularly schizophrenia. Replication and functional studies of these findings are warranted.

## Introduction

Major depressive disorder (MDD) is a serious psychiatric illness with a lifetime prevalence of ~13%.^1^ While several types of antidepressant medications have been shown to have beneficial effects for MDD symptoms, selection of the most appropriate medication for individual patients continues to be a challenge. Selective serotonin reuptake inhibitors (SSRIs) are the most commonly used medication class for MDD;^2, 3^ however, response to SSRI treatment varies considerably between patients, and it is widely recognized that identification of pharmacogenetic predictors of drug response has great potential to improve the treatment of MDD.^4^

Several genome-wide association studies (GWAS) of SSRI treatment outcomes have been performed.^5, 6, 7, 8, 9^ None of these studies have identified genetic variants that were associated with treatment outcomes at a genome-wide statistically significant level. Moreover, top findings from these studies have not been replicated in independent samples. Nevertheless, analysis of antidepressant response data using a mixed linear model approach to estimate the proportion of phenotypic variance explained by genome-wide single-nucleotide polymorphisms (SNPs, the GREML approach) implemented in the Genome-Wide Complex Trait Analysis package^10^ has demonstrated that common genetic variants explain a considerable proportion of individual differences in antidepressant response,^11^ providing compelling motivation for further pharmacogenomic studies.

Prior pharmacogenomic studies of antidepressant response have shown that GWAS have low power for discovery of relevant variants for this highly complex trait. Thus, large samples, such as those arising from the formation of collaborative consortia, will be necessary to make further progress in this field.^12, 13^ Taking this approach, Tansey *et al.*^13^ described a pharmacogenomic analysis of data arising from the NEWMEDS consortium, which includes response to serotonergic and noradrenergic antidepressants in over 2000 European-ancestry individuals with MDD. The analyses, which also included a meta-analysis with data from the Sequenced Treatment Alternatives to Relieve Depression (STAR*D) study,^14^ did not identify any common genetic variants associated with antidepressant response at a genome-wide significant level. Another recent meta-analysis combined the results of three prior studies to search for genetic variation associated with remission following treatment with antidepressants, and with SSRIs (escitalopram or citalopram) specifically.^12^ These analyses failed to replicate the single-study results in the pooled analysis. However, the analysis of the entire sample (all antidepressants) resulted in one genome-wide significant association of a SNP located in an intronic region of the myosin X (*MYO10*) gene. The analysis of the SSRI-treated subset revealed an association between early SSRI response (within 2 weeks of treatment) and a SNP in an intergenic region on chromosome 5.

The International SSRI Pharmacogenomics Consortium (ISPC) was established to investigate the genetic factors contributing to variable response to SSRIs. Because a variety of SSRIs were used in the studies contributed to the consortium, this sample provides the opportunity to identify genes that contribute to the pharmacodynamic, though not pharmacokinetic, effects of these medications. This is the first report of findings from a GWAS of SSRI treatment response based on the ISPC sample. Data from two prior GWAS of SSRI response, the Mayo Clinic Pharmacogenomic Research Network Antidepressant Medication Pharmacogenomics Study (PGRN-AMPS)^7, 15^ and the STAR*D study,^14^ were used for replication analysis, and a meta-analysis of the three studies was performed.

## Materials and methods

### Study design and samples

The ISPC initially included eight member sites (Supplementary Table S1), with one site subsequently excluded because of unavailable clinical data. The seven studies included in the analyses represent five countries from Europe, North America and Asia, with 2/3 of the sample being Asian (Taiwan, Thailand and Japan) and 1/3 being of European descent (Germany and the United States). All sites included in analyses had approval to participate in the consortium from their local ethical review board.

In total, 1149 DNA samples and clinical data for 998 subjects were contributed. Demographic and clinical data provided by individual sites were curated (that is, collected, formatted and subjected to quality control) by staff at the Pharmacogenetics and Pharmacogenomics Knowledge Base (PharmGKB, www.pharmgkb.org). Supplementary Table S2 lists the collected data, which included information on factors previously shown to be associated with antidepressant therapy response. These data included demographic characteristics, socioeconomic data, depression history, co-occurring diseases, antidepressant medication, dose and compliance, use of concomitant medications, rating scores of the individual items of the Hamilton Rating Scale for Depression (HRSD) at baseline and follow-up visits, side effects (if reported) and possible dose or antidepressant change at follow-up visits. Several subjects that did not receive SSRIs during the study period were removed from analysis.

A description of each contributing study, including the medications used in the study, is included in Supplementary Table S1, and key features of the samples used in the analyses are summarized in Table 1. The pharmacogenomic analysis focused on treatment outcomes at 4 weeks, as this time point was common to all participating studies. Prior studies have shown that early response in depression is strongly correlated with late response, which supports our choice of 4 weeks of observation period.^16, 17, 18^ However, because 4 weeks is considered too early to observe complete remission, our analyses focused on percentage reduction in HRSD score and response (defined as ⩾50% reduction in HRSD score) rather than remission, as described in the Statistical analysis section.

### Genotyping, quality control and imputation

All DNA samples (*N*=1149) were shipped to Mayo Clinic, Rochester, MN, USA for storage and plating, and were genotyped at the RIKEN Center of Integrative Medical Sciences (Yokohama, Japan) using Illumina HumanOmniExpressExome BeadChips. Three samples failed genotyping. Quality control assessments of the genetic data were performed as described in the Supplementary Materials, and structure analysis^19^ was used to infer ancestry using the genome-wide SNP data (Supplementary Figure S1), leading to removal of 16 subjects (11 related, 1 gender mismatch, 4 race outliers; see Supplementary Materials for details). As discussed in the Supplementary Methods section, there was clear population structure between sites, but without evidence of residual population stratification within sites. We verified that after adjustment for site, no further adjustment for ancestry via principal components was necessary. Thus, because site provided a good surrogate measure for ancestry, subsequent analyses were adjusted for site without further adjustment for population structure. After quality control, 631 765 genotyped SNPs remained for analysis. Genotypes at unobserved SNPs with minor allele frequency >0.01 in the reference population were imputed using Beagle (v3.3.1)^20^ with the 1000 genomes cosmopolitan reference panel. After removing markers with poor imputation quality (dosage *R*^2^<0.3), ~7 million imputed markers were available for analysis.

### Statistical analysis

The pharmacogenomic analyses focused on treatment outcomes at 4 weeks, as this time point was common to all participating studies. GWA analyses were performed for two phenotypes: ‘% change in HRSD-17 score' (%ΔHRSD defined as the change in HRSD-17 score divided by the baseline score) and ‘response' (defined as ⩾50% reduction in HRSD-17 score from baseline to 4-week visit). Because response is defined by dichotomizing %ΔHRSD, these two outcomes are highly correlated, and therefore results of analyses of the two outcomes are also correlated. However, the Spearman correlation of the test-statistics (or equivalent *P*-values) from analyses of these two outcomes is not very high (0.50 in the ISPC data), indicating that analyses of these two correlated outcomes can detect associations with different SNPs. Thus, analyses of both outcomes are presented.

The analysis included subjects with HRSD-17 data at baseline and week 4 (one entire site was removed due to missing clinical information), and was restricted to subjects with a baseline HRSD-17 score ⩾10. Table 1 shows the demographic and clinical characteristics of the final sample used for the GWA analyses (865 subjects from seven sites). Distributions of baseline HRSD-17, sex and age differed significantly among sites. All GWA analyses were adjusted for age and sex, in addition to site. However, because age and sex were not statistically significant predictors of treatment outcomes after adjustment for site, we also repeated the GWA analyses without age and sex adjustment. The results were virtually identical, and only the age- and sex-adjusted results are presented.

For the GWA analysis of %ΔHRSD, the association of each SNP with the outcome was tested using a linear regression model, with the SNP (coded as 0, 1, 2) and the covariates (age, sex and site) as independent variables and %ΔHRSD as the dependent variable. Because the distribution of %ΔHRSD demonstrated departures from normality, a van der Warden transformation was applied before analysis. For the binary outcome (response), logistic regression was used to test for SNP effects, while accounting for the covariate effects.

Analyses were performed in R (http://www.R-project.org), SAS (SAS Institute, Cary, NC, USA) and PLINK.^21^ *P*-values <5 × 10^−^^8^ were considered statistically significant at the genome-wide level.

### Replication of ISPC findings using PGRN-AMPS and STAR*D data

SNPs with the smallest *P*-values in each of the top 10 association regions in the two primary analyses (that is, top SNP in each region) were tested for association with equivalent outcomes in two independent samples: the PGRN-AMPS^15^ and STAR*D.^14^ GWA analyses utilizing these two samples are published,^5, 7^ and both data sets are available through controlled access via the database for Genotypes and Phenotypes (dbGaP; http://www.ncbi.nlm.nih.gov/gap).

Both the PGRN-AMPS and STAR*D evaluated depression outcomes following treatment with SSRIs. Although the PGRN-AMPS utilized both the HRSD and the 16-item Quick Inventory of Depressive Symptomatology (QIDS-C16) to measure depression symptoms at baseline and follow-up, STAR*D used primarily QIDS-C16 during the follow-up visits. Thus, replication analyses in the PGRN-AMPS data used the same treatment outcome definitions as the analysis of the ISPC data (based on HRSD-17), whereas replication analyses in the STAR*D data used equivalent %change and response outcomes definitions based on the QIDS-C16.

Analyses of the replication samples resembled those of the ISPC sample, using linear regression for quantitative outcomes and logistic regression for binary outcomes. Details of the quality control analyses in PGRN-AMPS have been described previously.^7^ To control for possible remaining population structure, analyses of the predominantly Caucasian PGRN-AMPS sample were adjusted for the first four eigenvectors derived from genome-wide SNP data thinned on the basis of linkage disequilibrium. After quality control filters (based on Garriock *et al.*,^5^ and removing 25 individuals from related pairs), genetic data were available for 1887 individuals from STAR*D; our analyses used data from 1529 subjects that had both baseline and 4-week QIDS-C16 measurements. Because the STAR*D sample included individuals of multiple ancestral backgrounds, analyses of this sample were adjusted for the first seven eigenvectors constructed from genome-wide SNP data, calculated in the same way as in the PGRN-AMPS.

### Meta-analyses of ISPC, PGRN-AMPS and STAR*D data

We also performed GWA analyses on the combined ISPC+AMPS sample, followed by a fixed-effects meta-analysis to combine the ISPC+AMPS GWA results with STAR*D GWA results (total sample size of 2394). Estimated effect sizes from the two analyses were combined by averaging the estimates weighted by the inverse of their variances. The combined estimates were then tested by a two-sided *Z*-test using a pooled standard error.

### Replication of results from prior genome-wide meta-analyses of antidepressant response

Finally, we used our sample to attempt replication of top findings from two recent large GWA studies of antidepressant response.^12, 13^ In particular, we attempted to replicate association with two SNPs that reached genome-wide significance in a published meta-analysis of GENDEP, MARS and STAR*D data^12^ (rs17651119 in *MYO10*, which was associated with 12-week percentage improvement in the entire sample, and the intergenic SNP rs12054895 that was associated with 2-week improvement in the SSRI-treated subset), as well as six other SNPs with suggestive evidence of association with one of the treatment outcomes (*P*<1.0E−06). Because this meta-analysis included the STAR*D sample, we evaluated replication of these prior findings in the ISPC sample and the combined ISPC plus PGRN-AMPS sample, but not in our meta-analysis that included STAR*D as that would not constitute an independent replication. We also investigated six SNPs with *P*<5.0E−06 reported by Tansey *et al.*^13^ for the overall analysis or the analysis of subjects treated with serotonergic antidepressants. For the SNPs reported by Tansey *et al.*, we evaluated replication in our ISPC sample, as well as in our ISPC-AMPS-STAR*D meta-analysis.

## Results

**Figure 1:**
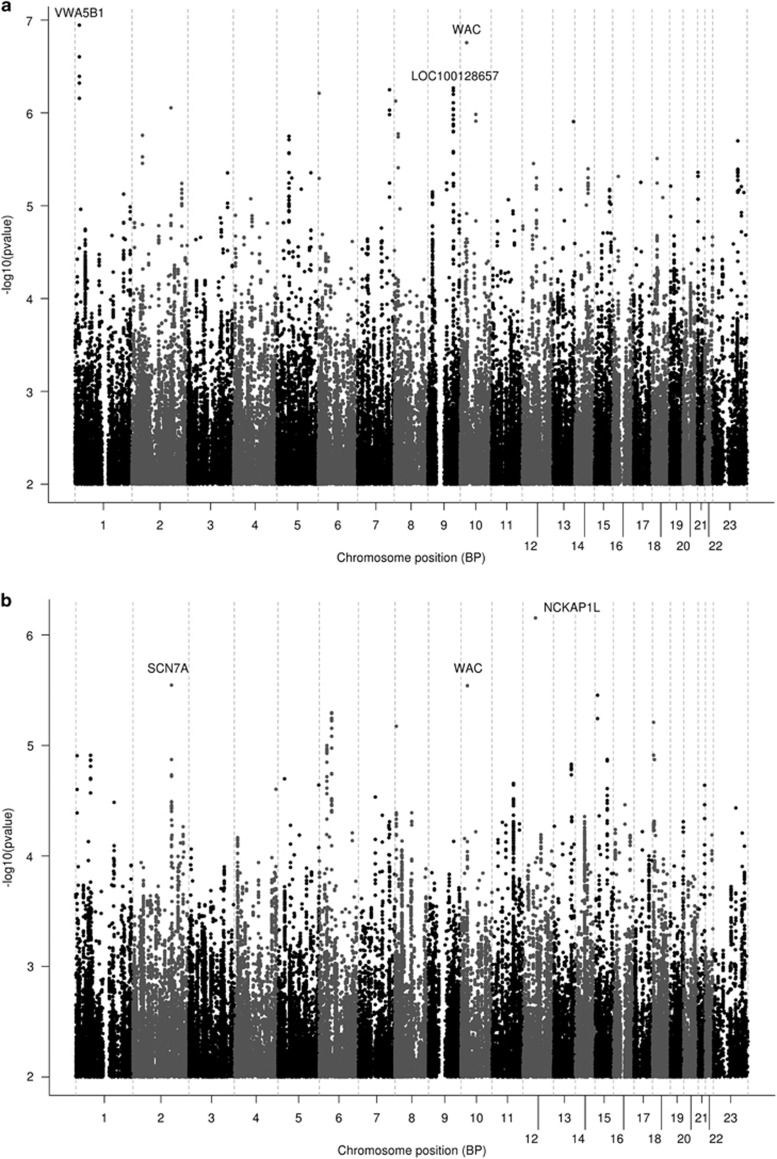
Manhattan plots showing genome-wide association results of the two outcome variables in the ISPC data analysis: (**a**) %ΔHRSD (**b**) response. HRSD, Hamilton Rating Scale for Depression; ISPC, International SSRI Pharmacogenomics Consortium.

Out of 865 individuals with baseline and 4-week HRSD-17 scores (baseline mean (s.d.)=22.2 (5.5); 4-week mean (s.d.)=11.9 (6.1)), 416 (48%) were responders (Table 1). Figure 1 shows the Manhattan plots for the association of genotyped and imputed SNPs with the two clinical outcomes (%ΔHRSD and response), with the corresponding QQ plots shown in Supplementary Figure S2. The top 10 genotyped SNP associations (smallest *P*-values) for each outcome are listed in Table 2; none of these associations reached genome-wide significance. Top results for %ΔHRSD included SNPs in the genes *MCPH1* (microcephalin 1) and *STK39* (serine threonine kinase 39). Top results for response included *MCPH1*, *PPIAP14* (peptidylprolyl isomerase A (cyclophilin A) pseudogene 14), *SCN7A* (sodium channel, type 7, alpha subunit), *BRD2* (bromodomain containing 2) and *HPRTP4* (hypoxanthine phosphoribosyltransferase pseudogene 4).

The top 10 SNP association regions including imputed SNPs are shown in Supplementary Table S3, where for each association region, the SNP with the smallest *P*-value is listed. The top association signal in the analysis of imputed SNP data was a low frequency SNP, rs56058016, in the von Willebrand factor A domain containing 5B1 gene (*VWA5B1*) with *P*=1.1E−07 for association with %ΔHRSD. Other new signals in the analysis of imputed SNPs, not reaching genome-wide significance, included *CTNNA3* (catenin alpha 3) for %ΔHRSD and *RYR3* (ryanodine receptor 3) for response. None of the top associations were replicated in the PGRN-AMPS or STAR*D analyses (Supplementary Table S3). Replication of the top association signal in *VWA5B1* could not be tested as this SNP was not imputed with adequate quality in the PGRN-AMPS and STAR*D data sets.

**Figure 2:**
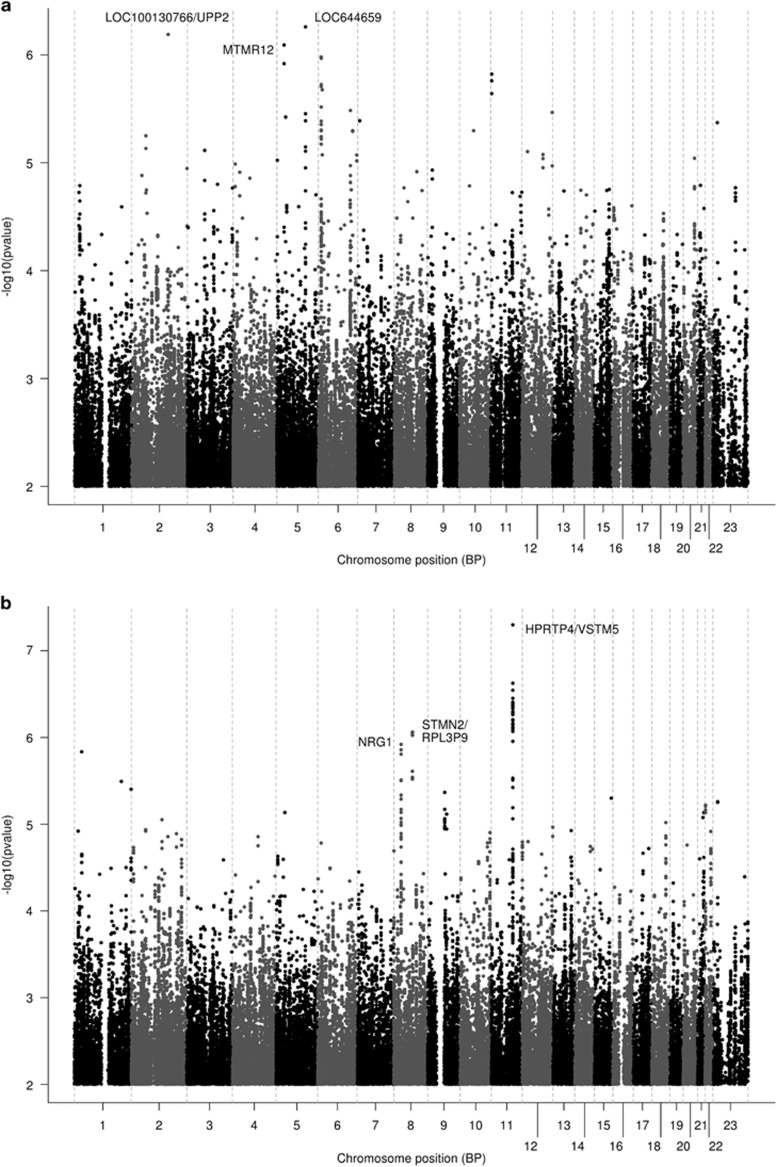
Manhattan plots showing genome-wide association results for the two outcome variables in the meta-analysis of ISPC, AMPS and STAR*D data. (**a**) %Δ depression score, (**b**) response. AMPS, Antidepressant Medication Pharmacogenomics Study; ISPC, International SSRI Pharmacogenomics Consortium; STAR*D, Sequenced Treatment Alternatives to Relieve Depression.

Results of the meta-analysis of the three data sets are shown in Figure 2 (Manhattan plots), Supplementary Figure S3 (QQ plots) and Table 3 (top 10 association SNPs for each outcome). In the meta-analysis of response, one SNP approached genome-wide significance (Table 3; rs2456568, *P*=5.0E−08). This SNP lies 3' downstream of the pseudogene *HPRTP4,* with other SNPs in this region with *P*-values <10^−6^ being located 5' upstream of the gene *VSTM5* (V-set and transmembrane domain containing 5; Supplementary Figure S4). Other notable top association regions for response include the 5' upstream region of the neuregulin gene, *NRG1* (Figure 2b and Supplementary Figure S5). Top association signals (*P*<1.0E−06) for %change in depression score include SNPs in the *MTMR12* (myotubularin-related protein 12) gene (Table 3).

Supplementary Table S4 shows results in the ISPC sample and the ISPC/PGRN-AMPS/STAR*D meta-analysis for SNPs selected on the basis of two recent large pharmacogenomic GWAS of antidepressant response.^12, 13^ Although the observed associations are not significant after correction for multiple testing of SNPs selected for these replication analyses, we observed marginally significant evidence of association of SNP rs11624702 in *MDGA2* (MAM domain containing glycosylphosphatidylinositol anchor 2) with response in the ISPC alone (*P*=0.061) and in the combined ISPC-AMPS-STAR*D meta-analysis (*P*=0.029), with the minor allele being associated with lower odds of response (that is, worse clinical outcomes). Nominally significant association was also found for SNP rs7174755 in *ITGA11* (integrin, alpha 11) in the combined ISPC+AMPS sample (*P*=0.010 for association with %ΔHRSD; *P*=0.025 for response). We also observed nominally significant association of the minor allele at SNP rs2546057 in *KCTD15* (potassium channel tetramerization domain containing 15) with higher odds of response (better clinical outcome) in the ISPC sample, but this association did not remain significant in the combined ISPC+AMPS analysis.

## Discussion

Genome-wide analyses of data from the ISPC presented here did not detect SNPs significantly associated with %ΔHRSD or response, and analyses of the PGRN-AMPS and STAR*D data did not replicate the top association signals from the analysis of the ISPC data. The top association signal was obtained for a set of imputed SNPs in the *VWA5B1* gene (*P*=1.1E−07). This result should be interpreted cautiously as this is a rare imputed SNP. A meta-analysis that included the ISPC, PGRN-AMPS and STAR*D data revealed a SNP in the region of the *HPRTP4* and *VSTM5* genes that approached genome-wide significance for association with response (*P*=5.0E−08). Replication of this association signal is warranted, before further functional follow-up. Moreover, several of the top association signals that did not achieve genome-wide significance were in genes of biological interest, most notably NRG1, which merits further investigation into their potential role in depression and antidepressant response.

The third highest association signal in our meta-analysis of response locates to the promoter region of the neurotrophic factor neuregulin-1 (*NRG1*) gene. Neuregulin is involved in many aspects of brain development, including neuronal maturation, and variations in *NRG1* are associated with risk for mental disorders including schizophrenia.^22, 23^ In fact, the SNP with strongest evidence of association with SSRI treatment outcomes identified here (rs10954808) is in linkage disequilibrium with rs7014762 (*r*^2^=0.80 in 1000 genomes CEU sample; *r*^2^=0.47 in 1000 genomes CHB/JPT sample), which was previously shown to be associated with a bipolar phenotype characterized by excellent recovery between episodes and no mood incongruent features.^24^ Another SNP in relatively high linkage disequilibrium with rs10954808 (rs4281084, *r*^2^=0.85 in 1000 genomes CEU sample, *r*^2^=0.61 in 1000 genomes CHB/JPT sample) was reported to be associated with transition to psychosis in at-risk individuals.^25^

Although NRG1 has been implicated in various psychiatric traits, this is, to the best of our knowledge, the first human pharmacogenomics study suggesting it may have a role in antidepressant treatment response. NRG1-ErbB4 signaling has a key role in the modulation of synaptic plasticity through regulating neurotransmission, and neuregulin was suggested to be a biomarker of MDD.^26^ In a recent animal model study, subchronic peripheral neuregulin-1beta administration increased ventral hippocampal neurogenesis and induced antidepressant-like behavior.^27^ Another recent study demonstrated that downregulation of NRG1-ErbB4 signaling in parvalbumin interneurons in the rat brain may contribute to the antidepressant properties of ketamine.^28^ In this study, pretreatment with NRG1 abolished both ketamine's antidepressant effects and ketamine-induced reduction in p-ErbB4, parvalbumin, 67-kDA isoform of glutamic acid decarboxylase (GAD67) and gamma-aminobutyric acid levels and increase in glutamate levels. Our results indicate that genetic variation in *NRG1* may also influence the antidepressant effects of SSRIs.

Other SSRI-response pharmacogenomics candidate genes arising from our study, including *MCPH1*, *RYR3*, *STK39*, *CTNNA3* and *PPEF2*, should be further investigated. Mutations in the human microcephalin gene *MCPH1* cause the autosomal recessive disorder primary microcephaly, characterized by a congenital reduction of brain size particularly in the cerebral cortex.^29^ A reduction in brain size could result from increased cell death or alternatively from a diminished self-renewal potential of neuroprogenitors. A recent report provided evidence that MCPH1 controls neuroprogenitor entry into mitosis.^30^ Neuroprogenitor cell proliferation is an early event of neurogenesis, a process by which new neurons are continuously generated and incorporated into the nervous system. Recent research has suggested that hippocampal neurogenesis may have a key role in antidepressant action.^31, 32^ A recent genetic study in a Japanese population found that *MCPH1* genetic variation is associated with automatic thoughts (may be risk factors for depression and anxiety) evaluated by the Depression and Anxiety Cognition Scale, a Japanese psychological questionnaire.^33^ *MCPH1* has also been implicated in autism and schizophrenia.^34, 35^

Ryanodine receptor 3, a protein that in humans is encoded by the *RYR3* gene, is normally enriched in hippocampal area CA1,^36^ suggesting a specialized role of this receptor in this area critical for depression and antidepressant action. Ryanodine receptors (RyR) increase activity-dependent calcium influx via calcium-induced calcium release. Calcium signals activate postsynaptic pathways in hippocampal neurons that underlie synaptic plasticity, learning and memory. Studies suggest that RyR2 and RyR3 isoforms have key roles in these processes.^37, 38^

The *STK39* gene encodes a serine/threonine kinase. The protein is localized to both the cytoplasm and the nucleus, may act as a mediator of cell stress response^39^ and has been implicated in autism.^40^ A prior GWAS suggested that genetic variation in *CTNNA3* (catenin alpha 3) is associated with antidepressant treatment-emergent suicidal ideation.^41^ Variants in *CTNNA3* have also been suggested to have a role in autism and schizophrenia.^31, 42^ Finally, *PPEF2*, a calmodulin-binding protein phosphatase, has been shown to influence mGluR5 levels and prior evidence suggests it may be involved in schizophrenia.^43^

We investigated the association of SSRI response and %ΔHRSD with SNPs with strongest evidence for association with treatment outcomes in the two recent large collaborative pharmacogenomics studies of antidepressants.^12, 13^ Consistent with earlier studies, which have shown difficulties with replication of pharmacogenomic predictors of antidepressant treatment outcomes, our analyses did not replicate the prior results, with the exception of nominal evidence of association for SNP rs11624702 in *MDGA2*, and rs2546057 in *KCTD15*. In particular, our analyses provided nominally significant evidence of association of SNP rs11624702 in *MDGA2* with response in the combined ISPC-AMPS-STAR*D meta-analysis. Previously, suggestive evidence of association of this SNP with antidepressant treatment outcomes was demonstrated in the genome-wide analysis of the NEWMEDS consortium sample (*P*=4.08 × 10^−^^6^).^13^ Other studies have also implicated this gene in depression-related traits and response to antidepressants. In particular, a genome-wide association study of SSRI/SNRI-induced sexual dysfunction in a Japanese cohort identified 11 *MDGA2* SNPs that were significantly associated with the outcome at a genome-wide significant level.^44^ Furthermore, *MDGA2* was identified as a potential neuroticism-related gene in a genome-wide association study,^45^ a finding that was subsequently replicated and extended by demonstrating association with a fatigability and asthenia subscale of harm avoidance.^46^ Thus, further investigation of the role of *MDGA2* in depression and SSRI-treatment response is warranted.

Although this study represents one of the largest pharmacogenomics studies of antidepressant response to date, we recognize it also has certain limitations. Because a variety of SSRIs were used in the studies contributed to the consortium, this sample provides the opportunity to identify genes that contribute to the pharmacodynamic, but not pharmacokinetic, effects of these medications. Another limitation of the presented analysis is the short time period of follow-up, as generally, 6–8 weeks is considered necessary for investigating full response. However, a 4-week period is sufficient for detecting a change in most patients, and therefore analyses of the 4-week outcomes were focused solely on relative symptom change and response.

As in all studies of patients with major depression, heterogeneity between individual patients likely limits the power of genetic analyses. Differences in samples across sites, including differences in the patient populations and treatments, further increase this phenotypic heterogeneity. Thus, the overall patient sample is likely to include subgroups with different susceptibility factors for clinical depression. These might involve endogenous, neurobiological factors, temporal stress factors and other environmental factors, such as those of cultural or dietary origin. Investigation of more homogeneous patient subsamples, such as severe depression or depression with melancholic or predominantly anxious features, may lead to additional insights despite reduced sample sizes. The fact that this study included a large Asian sample is a strength, as we believe there are no prior large pharmacogenomics studies of antidepressants in Asian populations. However, ethnic heterogeneity of the sample may also have reduced power of our GWA analyses, particularly due to differences in SNP allele frequencies. The possibility that in different ethnic populations different SNPs in the same genes may have functional impact on clinical phenotypes, implies that gene-level and pathway-level, rather than SNP-level, analyses may be more powerful in racially diverse samples. Such analyses, as well as an in-depth investigation of potential differences in genomic treatment outcome predictors across racial groups, will be the focus of subsequent publications.

The lack of replicated predictors of treatment response in published pharmacogenomics studies of antidepressants has been disappointing, and has prompted questions regarding how future research in this area should proceed. Recent studies have demonstrated that very large sample sizes are needed to achieve the power to identify genetic predictors of complex psychiatric traits,^47^ and the same is likely to be true for antidepressant response. However, other avenues must be explored to increase power of these studies. A high degree of clinical heterogeneity in samples of treated patients, as well as the broad definition of treatment outcomes in most prior pharmacogenomics studies of antidepressant response, are likely factors contributing to the lack of significant and replicable findings. Specifically, clinical subtypes of depression may respond differently to treatment with certain medications, and medication response may be modified by different genetic variants for these clinical subtypes. Moreover, treatment outcomes in pharmacogenomic studies of MDD are usually limited to overall improvement in depression severity as measured by scales such as the HRSD. However, specific genes may impact particular aspects of drug response that contribute to the total HRSD score (for example, certain types of mood changes, changes in sleep or appetite/weight and so on). Studies that focus on more narrowly defined groups of patients, or investigate specific aspects of treatment response, may thus provide additional insights into genetic predictors of response to antidepressants.

In conclusion, the present findings provide little evidence of specific genetic factors that would markedly affect the clinical response to SSRI treatment in major depression. Also, similar to prior efforts, the results of our meta-analyses showed a lack of robust treatment outcome predictors. Nevertheless, novel biological targets for further investigation have been identified. In particular, some of the associations found (such as NRG1 or MCPH1) might reflect the important role of neuronal renewal among the mechanisms of antidepressant response.^48^ There is a need to replicate the top association signals with larger samples. There is also a need for closer exploration of genes showing the most marked associations in more refined and homogeneous patient subsamples, such as severe depression or depression with melancholic or predominantly anxious features.

## Figures and Tables

**Table 1:** Demographic and clinical characteristics of the samples from individual ISPC member sites included in the genetic analysis

*Characteristic*	*Study 1*^a^	*Study 2*^a^	*Study 4*^a^	*Study 5*^a^	*Study 6*^a^	*Study 7*^a^	*Study 8*^a^	*Total/average*
Country	Taiwan	Taiwan	Germany	Germany	USA	Thailand	Japan	—
Number of subjects in GWAS	177	245	50	58	190	24	121	865
Age, mean (s.d.)	47.1 (15.2)	41.4 (13.7)	50.1 (17.1)	46.3 (13.6)	39.4 (13.6)	44.6 (10.7)	46.2 (15.4)	43.7 (14.7)
Sex, *N* (%) female	97 (54.8%)	201 (82.0%)	32 (64.0%)	43 (74.1%)	116 (61.1%)	15 (62.5%)	57 (47.1%)	561 (64.9%)
Baseline HRSD-17 score, mean (s.d.)	26.4 (4.05)	21.3 (4.18)	22.6 (7.55)	24.2 (6.10)	21.0 (4.87)	15.0 (3.58)	20.0 (5.68)	22.2 (5.53)
4-Week HRSD-17 score, mean (s.d.)	13.6 (5.33)	12.4 (5.17)	8.84 (7.90)	13.6 (7.81)	12.0 (6.05)	7.58 (6.79)	9.37 (5.73)	11.9 (6.12)
Remitters at 4 weeks, *N* (%)	20 (11.3%)	47 (19.2%)	26 (52.0%)	17 (29.3%)	49 (25.8%)	16 (66.7%)	51 (42.1%)	226 (26.1%)
Responders at 4 weeks, *N* (%)	89 (50.3%)	97 (39.6%)	34 (68.0%)	24 (41.4%)	82 (43.2%)	15 (62.5%)	75 (62.0%)	416 (48.1%)

Abbreviations: GWAS, genome-wide association study; HRSD, Hamilton Rating Scale for Depression; ISPC, International SSRI Pharmacogenomics Consortium.

a Study numbers correspond to the contributing studies described in Supplementary Table S1.

**Table 2:** Top clinical outcome association results among the genotyped SNPs in the ISPC sample

*Outcome measure*	*SNP*	*CHR*	*BP*	*Nearest gene(s)*	*MA*	*CA*	*MAF*	*C-Frq*	*A-Frq*	*Beta/OR*	P
%ΔHRSD	rs9328202	6	3887339	RPS25P7	A	C	0.027	0.04	0.02	0.72	6.15E−07
	rs11989215	8	6395909	ANGPT2/MCPH1	G	A	0.360	0.36	0.36	0.24	7.46E−07
	rs16855294	2	169199695	STK39	A	C	0.416	0.60	0.32	−0.24	8.82E−07
	rs7802493	7	138699590	ZC3HAV1L	G	A	0.010	0.03	0	1.14	9.35E−07
	rs10512361	9	110974998	LOC100128657	G	A	0.064	0.18	0	−0.46	2.61E−06
	rs2453488	12	49255208	RND1/DDX23	A	G	0.115	0.27	0.03	−0.37	3.51E−06
	rs1438692	5	148659664	AFAP1L1	A	G	0.491	0.38	0.55	−0.21	4.42E−06
	rs239022	21	17701594	LINC00478	A	G	0.109	0.05	0.14	0.34	4.82E−06
	rs1470108	15	89153744	MIR7-2/MIR3529/MIR1179	A	C	0.216	0.35	0.15	−0.26	6.67E−06
	rs11811628	1	212756393	ATF3	A	G	0.013	0.04	0	0.96	7.50E−06
Response	rs11989215	8	6395909	MCPH1/ANGPT2	G	A	0.360	0.36	0.36	0.62	6.70E−06
	rs1466882	18	4970506	PPIAP14	C	A	0.466	0.52	0.44	1.55	1.23E−05
	rs13015447	2	167377978	SCN7A	A	C	0.443	0.63	0.35	1.57	1.34E−05
	rs2377898	18	8730053	SOGA2	A	G	0.456	0.47	0.45	0.65	1.34E−05
	rs17220479	6	32940815	BRD2	A	G	0.055	0.02	0.07	0.34	1.42E−05
	rs16871297	6	32950217	BRD2	G	A	0.055	0.02	0.07	0.34	1.42E−05
	rs12729349	1	65570028	MRPS21P1	A	G	0.135	0.08	0.16	0.52	2.02E−05
	rs2511398	11	93684809	HPRTP4	G	A	0.433	0.56	0.37	1.51	2.28E−05
	rs2218603	2	167472743	SCN7A	A	G	0.420	0.54	0.36	1.54	3.28E−05
	rs1596996	2	167453461	SCN7A	A	G	0.452	0.59	0.38	1.53	3.63E−05

Abbreviations: A-Frq, frequency of MA in Asian subset; Beta/OR, regression parameter estimates (beta) for SNP effect on quantitative trait outcome or odds ratio (OR) estimate for SNP effect on binary outcome; CA, common allele; C-Frq, frequency of MA in Caucasian subset; HRSD, Hamilton Rating Scale for Depression; ISPC, International SSRI Pharmacogenomics Consortium; MA, minor allele; MAF, minor allele frequency; SNP, single-nucleotide polymorphism.

**Table 3:** Top 10 association regions from the genome-wide association meta-analysis of the combined ISPC+AMPS sample and STAR*D (all race)

*Outcome measure*	*SNP*	*CHR*	*BP*	*Gene*	*ISPC+AMPS Beta/OR*	STAR*D Beta/OR	*Meta-analysis Beta/OR*	P
% Change in depression score	rs6889896	5	124699239	LOC644659	−0.17	−0.16	−0.17	5.49E−07
	rs35806662	2	158790425	LOC100130766	−0.18	−0.21	−0.20	6.47E−07
	rs73069924	5	32278233	MTMR12	−0.50	−0.42	−0.44	8.09E−07
	rs4615376	6	13071073	PHACTR1	−0.20	−0.13	−0.16	1.05E−06
	rs2566255	11	4605195	OR52I2/C11orf40	0.24	0.26	0.26	1.51E−06
	rs910039	6	17505944	CAP2	0.10	0.17	0.13	2.11E−06
	rs17068112	6	139250125	REPS1	0.19	0.37	0.26	3.28E−06
	rs7485210	12	131638853	LOC116437	0.18	0.10	0.14	3.42E−06
	rs74378198	5	38870908	OSMR	−0.69	−0.37	−0.43	3.77E−06
	rs4236420	7	9688109	PER4	0.10	0.17	0.13	4.08E−06
Response	rs2456568	11	93691332	HPRTP4	1.43	1.29	1.36	5.03E−08
	rs113889867	8	80556500	STMN2	0.67	0.42	0.51	8.68E−07
	rs10954808	8	31479623	NRG1	0.73	0.73	0.73	1.20E−06
	rs506546	1	34439294	CSMD2	0.70	0.83	0.76	1.46E−06
	rs74546197	1	205880179	SLC26A9	0.45	0.47	0.46	3.21E−06
	rs72772787	1	248016378	TRIM58	2.63	1.94	2.34	3.96E−06
	rs2043144	9	73950955	TRPM3	0.33	0.42	0.38	4.30E−06
	rs291028	15	95615786	LOC400456	0.61	0.60	0.61	5.02E−06
	rs7051085	23	23374977	PTCHD1	0.60	0.69	0.63	5.51E−06
	rs1210638	22	18981563	DGCR5	1.34	1.32	1.33	6.04E−06

Abbreviations: AMPS, Antidepressant Medication Pharmacogenomics Study; Beta/OR, regression parameter estimates (beta) for SNP effect on quantitative trait outcome or odds ratio (OR) estimate for SNP effect on binary outcome; ISPC, International SSRI Pharmacogenomics Consortium; SNP, single-nucleotide polymorphism; STAR*D, Sequenced Treatment Alternatives to Relieve Depression.

Rather than showing results for multiple SNPs in one region, only one (top) SNP is shown for each association region. There were other SNPs in these regions with suggesting evidence for association, for example, in the chromosome 11 region near HPRTP4, there were 34 SNPs with *P*<10^−6^.

## Notes

VA is a member of the advisory boards (past 5 years) for Astra-Zeneca, Eli Lilly, Lundbeck, Otsuka, Servier, Trommsdorff. BTB is a member of advisory board (past 5 years) of Lundbeck. RW and LW own stock in OneOme LLC. RBA and TEK are stockholders in Personalis. The remaining authors declare no conflict of interest.
