# The International SSRI Pharmacogenomics Consortium (ISPC): a genome-wide association study of antidepressant treatment response

**DOI:** 10.1038/tp.2016.187

**Published:** 2016-11-01

**Authors:** J M Biernacka, K Sangkuhl, G Jenkins, R M Whaley, P Barman, A Batzler, R B Altman, V Arolt, J Brockmöller, C H Chen, K Domschke, D K Hall-Flavin, C J Hong, A Illi, Y Ji, O Kampman, T Kinoshita, E Leinonen, Y J Liou, T Mushiroda, S Nonen, M K Skime, L Wang, B T Baune, M Kato, Y L Liu, V Praphanphoj, J C Stingl, S J Tsai, M Kubo, T E Klein, R Weinshilboum

**Correction to:** *Translational Psychiatry* (2015) **5**, e553; doi:10.1038/tp.2015.47; published online 21 April 2015

Following publication, the authors identified an error in the meta-analysis of ISPC, AMPS and STAR*D data. The error resulted from a failure to correctly harmonize alleles at some SNPs prior to meta-analyzing results from the three studies. This error had no impact on the primary analysis, which focused on the ISPC data alone (Table 2, Figure 1), but resulted in several incorrect signals being listed in Table 3 (meta-analysis results). The corrected Table 3 is shown below, along with a corrected Figure 2 (Manhattan plot of the meta-analysis). A new Supplementary File is available online. The corrections to the article are described below.

In the Abstract, the sentence ‘Top association results in the meta-analysis of response included single-nucleotide polymorphisms (SNPs) in the *HPRTP4* (hypoxanthine phosphoribosyltransferase pseudogene 4)/VSTM5 (V-set and transmembrane domain containing 5) region, which approached genome-wide significance (*P*=5.03E−08) and SNPs 5′ upstream of the neuregulin-1 gene, *NRG1* (*P*=1.20E−06)' should read ‘The top association result in the meta-analysis of response represents SNPs 5′ upstream of the neuregulin-1 gene, *NRG1* (*P*=1.20E−06).'

In the Results section, the following sentence should not have been included in the article: ‘In the meta-analysis of response, one SNP approached genome-wide significance (Table 3; rs2456568, *P*=5.0E−08). This SNP lies 3′ downstream of the pseudogene *HPRTP4*, with other SNPs in this region with *P*-values <10^−6^ being located 5′ upstream of the gene *VSTM5* (V-set and transmembrane domain containing 5; Supplementary Figure S4).' The subsequent sentence should not have begun with ‘Other' the corrected sentence is ‘Notable top association regions for response include the 5′ upstream region of the neuregulin gene, *NRG1* (Figure 2b and Supplementary Figure S4).'

Also in the Results section, the following sentence should have appeared with the two changes shown in square brackets: ‘Although the observed associations are not significant after correction for multiple testing of SNPs selected for these replication analyses, we observed marginally significant evidence of association of SNP rs11624702 in *MDGA2* (MAM domain containing glycosylphosphatidylinositol anchor 2) with response in the ISPC alone (*P*=0.061) and [‘and' should read ‘but not'] in the combined ISPC-AMPS-STAR*D meta-analysis (*P*=0.029) [‘0.029' should read ‘0.36'], with the minor allele being associated with lower odds of response (that is, worse clinical outcomes)'.

In the Discussion section, the statement ‘A meta-analysis that included the ISPC, PGRN-AMPS and STAR*D data revealed a SNP in the region of the HPRTP4 and VSTM5 genes that approached genome-wide significance for association with response (p=5.0E-08). Replication of this association signal is warranted, prior to further functional follow-up. Moreover, several of the top association signals that did not achieve genome-wide significance were in genes of biological interest, most notably NRG1, which merits further investigation into their potential role in depression and antidepressant response.' should read ‘A meta-analysis that included the ISPC, PGRN-AMPS and STAR*D data revealed several association signals that did not achieve genome-wide significance but were in genes of biological interest, most notably NRG1, which merits further investigation into their potential role in depression and antidepressant response.'

Also in the Discussion section, the following sentence should not have been included: ‘In particular, our analyses provided nominally significant evidence of association of SNP rs11624702 in *MDGA2* with response in the combined ISPC-AMPS-STAR*D meta-analysis.'

## Figures and Tables

**Figure 2:**
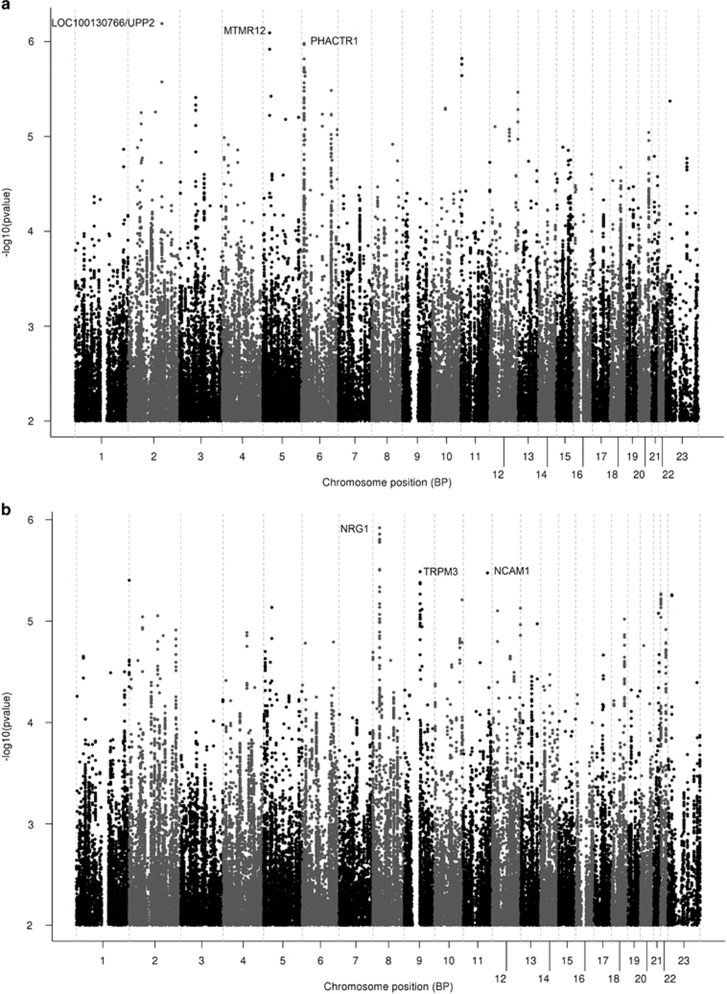
Manhattan plots showing genome-wide association results for the two outcome variables in the meta-analysis of ISPC, AMPS and STAR*D data. (a) %Δ depression score, (b) response. AMPS, Antidepressant Medication Pharmacogenomics Study; ISPC, International SSRI Pharmacogenomics Consortium; STAR*D, Sequenced Treatment Alternatives to Relieve Depression.

**Table 3:** Top 10 association regions from the genome-wide association meta-analysis of the combined ISPC+AMPS sample and STAR*D (all race)

*Outcome measure*	*SNP*	*CHR*	*BP*	*Gene*	*A1/A2*	*ISPC+AMPS (beta/OR)*	STAR*D (beta/OR)	*Meta-analysis (beta/OR)*	P
%Change in depression score	rs35806662	2	158790425	LOC100130766/UPP2/ACVR1	G/A	−0.18	−0.21	−0.20	6.47E−07
	rs73069924	5	32278233	MTMR12	G/A	−0.50	−0.42	−0.44	8.09E−07
	rs4615376	6	13071073	PHACTR1	G/A	−0.20	−0.13	−0.16	1.05E−06
	rs2566255	11	4605195	OR52I2/C11orf40	G/C	0.24	0.26	0.26	1.51E−06
	rs910039	6	17505944	CAP2	A/G	−0.10	−0.17	−0.13	2.11E−06
	rs17068112	6	139250125	REPS1	G/C	0.19	0.37	0.26	3.28E−06
	rs7485210	12	131638853	LOC116437/LINC01257/GPR133	A/C	0.18	0.10	0.14	3.42E−06
	rs74378198	5	38870908	OSMR	G/A	−0.69	−0.37	−0.43	3.77E−06
	rs10511037	3	74669078	CNTN3	G/A	−0.21	−0.14	−0.19	3.89E−06
	rs5904601	23	22016054	SMS/PHEX	C/T	0.15	0.13	0.14	4.24E−06
									
Response	rs10954808	8	31479623	NRG1	G/A	0.73	0.73	0.73	1.20E−06
	rs2309896	9	73959235	TRPM3	C/T	0.33	0.43	0.38	3.26E−06
	rs674246	11	113141684	NCAM1	C/T	1.28	1.38	1.33	3.35E−06
	rs72772787	1	248016378	TRIM58	T/C	2.63	1.94	2.34	3.96E−06
	rs2540651	22	18972331	DGCR5	A/G	1.37	1.28	1.33	5.39E−06
	rs7051085	23	23374977	PTCHD1	A/T	0.60	0.69	0.63	5.51E−06
	rs11018318	10	128774073	DOCK1	A/G	1.30	1.48	1.38	6.17E−06
	rs74378198	5	38870908	OSMR	G/A	3.96	2.19	2.40	7.34E−06
	rs4759855	12	131640425	LOC116437	G/T	0.67	0.67	0.67	7.47E−06
	rs116842540	9	82367951	TLE4	T/A	0.36	0.39	0.37	7.67E−06

Abbreviations: AMPS, Antidepressant Medication Pharmacogenomics Study; Beta/OR, regression parameter estimates (beta) for SNP effect on quantitative trait outcome or odds ratio (OR) estimate for SNP effect on binary outcome; ISPC, International SSRI Pharmacogenomics Consortium; SNP, single-nucleotide polymorphism; STAR*D, Sequenced Treatment Alternatives to Relieve Depression. There were other SNPs in these regions with suggestive evidence for association.

Note: Rather than showing results for multiple SNPs in one region, only one (top) SNP is shown for each association region.

