# Supplementary material for: The International SSRI Pharmacogenomics Consortium (ISPC): a genome-wide association study of antidepressant treatment response
Source: Transl Psychiatry. 2015 Apr 21;5(4):e553–. doi: 10.1038/tp.2015.47 (PMC4462610; doi:10.1038/tp.2015.47)
Supplement: Supplementary Information [file tp201547x1.docx]

**Supplemental Methods and Results**

**Sample Description**

A summary of the contributing studies is shown in Supplemental Table S1, and a brief description of each study follows. Supplemental Table S2 lists the variables that were collected, where available, for each subject included in the pooled dataset.

***Site 1: Taipei Veterans General Hospital, Taipei, Taiwan***

This sample has been used for a number of candidate gene pharmacogenetic studies ^[1-3](#_ENREF_1)^. Briefly, the case population was comprised of patients diagnosed with a current major depressive episode fulfilling DSM-IV criteria. Each diagnosis was made by one board-certificated and experienced psychiatrist in accordance with DSM-IV. Additional inclusion criteria were a minimum score of 18 on the 21-item HRSD, presence of depressive symptoms, and antidepressant-naive/free for at least 2 weeks prior to study commencement. Exclusion criteria were extra DSM-IV Axis I diagnoses, personality disorders, pregnancy, recent suicide attempt, and major medical and/or neurological disorders. The sample consisted entirely of ethnically Chinese adults. For the pharmacogenetic study, daily doses of fluoxetine or citalopram were given, starting at 20 mg/day; based on the clinical response the investigator could increase the dosage to 40 mg/day. No other psychotropic medications were permitted; however, anxiolytics were allowed for insomnia. Treatment efficacy was evaluated by the same investigator, blind to patient genotype, who administered the HRSD prior to treatment, in the 1st week, 4th week and 8th week.

***Site 2: Center for Neuropsychiatric Research, National Health Research Institutes, Zhunan, Taiwan***

This study was approved by the institutional review boards of the National Health Research Institutes and all participating clinics, with the requirement of written informed consent signed by each study participant. Patients were recruited from outpatient clinics of five hospitals in northern Taiwan. All participants were at least 18 years old and had a depressive episode at their baseline visit with a score of at least 14 on the HRSD-21. Patients were interviewed by board certified psychiatrists and trained research nurses. The clinical diagnosis was made according to DSM-IV criteria, using the Structured Clinical Interview for DSM-IV Axis-I disorders. Those with a primary or a comorbid diagnosis of schizophrenia, schizoaffective disorder, bipolar disorder, alcohol or substance dependence, dementia, or other significant medical conditions, and those who had been treated previously with paroxetine or escitalopram were excluded.

Before entering the study, patients needed to have completed a 7-day washout period for any earlier antidepressant treatments (12 days for fluoxetine). Patients were administered either escitalopram or paroxetine according to the judgment of study clinicians. In the escitalopram arm, patients received a daily fixed dose of 10 mg for the first 4 weeks, followed by flexible dosages of 10–30 mg/day on the basis of their clinical response over an 8-week treatment. In the paroxetine arm, patients received a daily fixed dose of 20 mg paroxetine for the first 4 weeks, and then a second 4-week period of flexible dosing (20–40 mg/day). No other psychotropic drugs were allowed during this period, except for 10 mg of zolpidem per night to treat insomnia (as needed, but for no more than four nights per week) and 1–2 mg of lorazepam per day to treat anxiety symptoms as needed. Study participants were assessed using the HRSD-21 and the Hamilton Rating Scale for Anxiety (Hamilton, 1959), at week 0 (baseline or the time of enrollment), 1, 2, 4, 6, and 8 of the continuous treatment period. Additional details about the study can be found in prior publications [^4-6^](#_ENREF_4)

***Site 4: Muenster University Hospital, Muenster, Germany***

Approval of the ethics committee of the University of Muenster, Muenster, Germany, and written informed consent from all subjects were obtained before commencement of any study procedures. Parts of the present sample have already been described in published genetic or pharmacogenetic studies targeting other gene systems ^[7-9](#_ENREF_7)^. Briefly, patients were ascertained from consecutive admissions for inpatient treatment at the Department of Psychiatry, University of Muenster, Germany, between 2004 and 2008. Patients’ lifetime diagnoses related to major depression and bipolar disorder were ascertained by the use of a structured clinical interview (SCID-I) according to the criteria of DSM-IV. Patients with schizoaffective disorders or comorbid substance abuse disorders, mental retardation, pregnancy and neurological, neurodegenerative disorders or other clinically unstable medical illnesses impairing psychiatric evaluation were not included in this analysis. Clinical course of depression was assessed using the HRSD-21 scale on a weekly basis. Patients were treated in a naturalistic setting with a variety of antidepressant medication according to doctor’s choice. None of the included patients had received electroconvulsive therapy within six months before the present investigation.

***Site 5: University of Göttingen, Germany and Federal Institute for Drugs and Medical Devices, Bonn, Germany***

The study was approved by the ethics committee of the Charité University Medical Centre of the Humboldt University and all patients gave their written informed consent to participate in the study. A convenience cohort of hospitalized patients with a diagnosis of major depression was recruited from three psychiatric hospitals in the central area of Berlin, Germany. The cohort has been described in prior publications ^[10-12](#_ENREF_10)^. Patients were followed during hospital stay for 3 weeks of antidepressant drug treatment. The diagnosis of major depression was made by the Mini International Neuropsychiatric Interview (M.I.N.I.), and severity of depression was rated by the HDRS-21. All patients had to receive at least one antidepressant drug during the three weeks of the observation period. If more than one antidepressant drug was prescribed, antidepressant drugs were regarded to contribute to response if medication was started at least one week prior to response assessment (HDRS-21) on day 21 and if it was not stopped before 3 days prior to severity assessment. Thus, all antidepressant drugs taken during the time frame of 3 days prior to first visit and 14 days after first visit were regarded to potentially contribute to drug response assessed on day 21.

***Site 6: Mayo Clinic, Rochester, Minnesota***

The patients for this protocol were recruited from the inpatient and outpatient practices in the Department of Psychiatry and Psychology in Rochester, Minnesota and Mayo Clinic Health System in Austin, Minnesota. Patients could also be referred to the study by their primary care physician at Mayo Clinic Rochester. Patients were eligible for the study if they were between the ages of 18 to 85 and had a diagnosis of non-psychotic MDD. Patients needed a score of ≥ 14 on the HRS-D17 to be eligible for the study. Patients who had medical contraindications to citalopram or escitalopram and those you previously failed to respond to an adequate course of treatment of citalopram or escitalopram were excluded. We also excluded patients with schizophrenia, schizoaffective disorder, bipolar I disorder, active substance abuse, active suicidal ideation, or who were pregnant or breast-feeding. Patients could not be on any other antidepressant medication during the course of the study. Trazodone, melatonin, and diphenhydramine could be used as rescue medications for insomnia. Benzodiazepines could be used for treatment of anxiety, and atomoxetine could be used for the treatment of attention deficit disorder. Study subjects that were currently on antipsychotic medications (e.g., typical and atypical antipsychotic drugs) and mood stabilizing agents (e.g., lithium, carbamazepine, valproate, lamotrigine, gabapentin, or other anticonvulsants) were not eligible for the study with the exception of those starting quetiapine after baseline. The protocol for this study has been described in greater detail elsewhere [^13^](#_ENREF_13)^,^ [^14^](#_ENREF_14); however, we emphasize that the subjects included in these prior publications were not part of the ISPC cohort. In particular, the subjects that were included in the previously-published PGRN-AMPS GWAS [^13^](#_ENREF_13) were not part of the ISPC, but rather were included in this study as one of the replication cohorts. New subjects subsequently enrolled at Mayo Clinic, who were not part of the AMPS cohort, were part of the ISPC data set.

***Site 7: Rajanukul Institute, Thailand***

Patients included in this group are selected from the “Pharmacogenetics study in major depressive disorder in Thai population” study, conducted during 2008-2011. The study was approved by the Ethical Review Committee for Research in Human Subjects, Department of Mental Health, the Ministry of Public Health, Thailand. Written informed consent was obtained from each participant.

The study was an observational, opened-label, one-arm clinical trial to detect genetic variants associated with fluoxetine response in patients with major depression. Participants, aged above 18 years old, diagnosed by a psychiatrist as having major depressive disorder, major depressive disorder with drug-induced mania, major depressive disorder on top dysthymia who had never received treatment or had stopped treatment more than 6 months prior were enrolled in the study. Patients with schizophrenia, bipolar disorder, schizoaffective disorder, substance-induced depression, serious physical illnesses, on steroid drug use, were excluded from the study. Patients who received concomitant treatment with stimulants, anticonvulsants, antipsychotics, alprazolam, other anti-depression (except for trazodone less than 200 mg for treating insomnia), or psychotherapy for treating depression, were discontinued from the study.

Relevant data was collected by trained clinical psychologists using the following tools: Diagnostic Interview for Genetics Study (Thai version), Family Interview for Genetics Study (Thai version), and Stressful Life Event (Thai version). Self-reporting Temperament and Character Inventory (TCI 240) was completed by the participant towards the end of the study. Hamilton Rating Scale for Depression (HRSD-17) (Thai version) and Global Rating of Side Effect Burden (Thai version) were used to collect data every two weeks during the 12 weeks of treatment. Dosage of fluoxetine was prescribed and adjusted according to the psychiatrist judgment to the clinical response.

***Site 8: Kansai Medical University, Osaka, Japan***

Patients included in this study participated in randomized controlled trials of antidepressants at the Kansai Medical University, Osaka ^[15-17](#_ENREF_15)^. Diagnosis of major depression was confirmed by the Structured Clinical Interview for DSM-IV Axis I Disorders (SCID-I). Exclusion criteria were as follows: clinically significant unstable medical illness, pregnancy, main psychiatric diagnosis other than major depression (including dementia, mental retardation, substance abuse, dysthymia, panic disorder, obsessive–compulsive disorder and generalized anxiety disorder), and electroconvulsive therapy within the previous 6 months. The study was approved by the ethical committee of the Kansai Medical University and Osaka University. Written informed consent was obtained from each subject after a detailed explanation of the study.

The diagnoses were made by two independent senior psychiatrists and confirmed by a third psychiatrist. All patients were evaluated at baseline and bi-weekly thereafter until week 6 using the 21-item HRSD administered by a trained senior psychiatrists blind to genetic data. Patients were either drug free or taking ineffective antidepressants and after ten days of washout, paroxetine (n = 68) or fluvoxamine (n = 54) was administered to reach therapeutic doses from days 8 to 11 until the end of trial (fluvoxamine: 150 mg/day; paroxetine: 40 mg/day). The type of prescribed SSRI remained unchanged throughout the study period.

**Methods: Genetic Data Quality Control and Imputation**

Quality control assessments included genotype concordance rates of duplicate samples and Mendelian inheritance checks for a CEPH trio of two parents and their child. For each single nucleotide polymorphism (SNP), minor allele frequency, call-rate and departure from Hardy-Weinberg equilibrium were evaluated. Observed call-rates, total heterozygosity and inbreeding coefficients were assessed for each subject using PLINK[^18^](#_ENREF_18). Sex-checks based on X-chromosome heterozygosity were performed, and tests for identity-by-descent (IBD) were used to identify potentially related subjects. Based on these quality control measures, 11 samples were removed due to relatedness (one subject was removed from each pair that appeared to have a relationship closer than first cousins), and one sample was removed due to a gender mismatch (reported gender was discrepant with X-chromosome genotype data). After exclusion of Y-chromosome, mitochondrial, and unplaced SNPs, as well as SNPs that failed genotyping, had minor allele frequency (MAF) < 0.01, or showed departures from Hardy-Weinberg Equilibrium (p<10^-6^ in Caucasian or Asian subset, or p<10^-4^ in both the Caucasian and Asian subset), 631,765 SNPs remained for analysis.

Structure (version 2.1)[^19^](#_ENREF_19) analysis was used to infer ancestry based on a subset of SNPs. First, SNPs on the X and Y chromosomes, as well as SNPs in 4 chromosomal regions that can have a strong influence on principal components of genome-wide SNP data (8p23, 2q21, 17q21.31, and HLA/6p21.32), were removed. The remaining SNPs were pruned based on LD using the snpgdsLDpruning function in the SNPrelate package in R, leading to about 73,000 SNPs that were used in subsequent Structure and Eigenstrat analyses. The Structure analysis demonstrated strong clustering of individuals by site (Supplemental Figure S1), with the exception of four outliers that did not cluster with other subjects from the same site. These four subjects were excluded from analysis. As there was clear population structure between sites, but without evidence of residual population stratification within sites, site provided a good surrogate measure for ancestry. Therefore, subsequent analyses were adjusted for site without further adjustment for ancestry.

**Supplemental Table S1**: ISPC Participating Sites and Study Description

| Study | Site | PI | Medication(s) | Study Duration | References |
| --- | --- | --- | --- | --- | --- |
| 1 | Taipei Veterans General Hospital, Taipei, Taiwan | Shih-Jen Tsai  Chen-Jee Hong  Ying-Jay Liou  Younger WY Yu  Tai-Jui Chen | fluoxetine, citalopram | 8 weeks | [^1-3^](#_ENREF_1) |
| 2 | Center for Neuropsychiatric Research, National Health Research Institutes, Miaoli County, Taiwan | Chia-Hui Chen  Yu-Li Liu | escitalopram, paroxetine | 8 weeks | [^4-6^](#_ENREF_4) |
| 3 | Tampere University Hospital, Finland | Ari Illi Esa Leinonen Olli Kampman | Fluoxetine Paroxetine Citalopram | 6 weeks | [^20^](#_ENREF_20) |
| 4 | Muenster University Hospital, Germany | Volker Arolt  Bernhard Baune,  Katharina Domschke | escitalopram, paroxetine and sertraline | 6 weeks | [^7-9^](#_ENREF_7) |
| 5 | University of Göttingen, Germany and Federal Institute for Drugs and Medical Devices, Bonn, Germany | Jürgen Brockmöller and Julia Stingl (former name Kirchheiner) | citalopram, escitalopram, paroxetine, sertraline, venlafaxine | 3 weeks observation in hospital | [^10-12^](#_ENREF_10) |
| 6 | Mayo Clinic, USA | David A. Mrazek  Joanna Biernacka  Richard Weinshilboum | citalopram, escitalopram | 8 weeks | [^21^](#_ENREF_21)^,^ [^22^](#_ENREF_22) |
| 7 | Rajanukul Institute, Thailand | Verayuth Praphanphoj | fluoxetine | 8 weeks |  |
| 8 | Kansai Medical University, Japan | Masaki Kato  Masataka Wakeno | paroxetine, fluvoxamine | 6 weeks | [^15-17^](#_ENREF_15) |

**Supplemental Table S2:** Data Collected from Participating Sites

| **Demographic Data** | |
| --- | --- |
| Gender | Male or female |
| Race | Self-reported information and racial categories used as defined by the U.S. Office of Management and Budget |
| Ethnicity | Self-reported information and racial categories used as defined by the U.S. Office of Management and Budget |
| Age | Age at time of consent in years |
| **Baseline Data** | |
| Height | Height in centimeter |
| Weight | Weight in kilogram |
| What is patient's current employment status? | Unemployed, 2=employed, 3=retired, 99=unknown |
| Is patient married or in a long-term relationship? | 1=yes, 0=no, 2=widow/widower, 99=unknown |
| Does patient live with spouse or life partner? | 1=yes, 0=no, 99=unknown |
| Age at onset of first episode of depression | years, 99=unknown |
| Number of depressive episodes until time of study enrollment | Number, 99=unknown |
| Number of manic episodes until time of study enrollment | Number, 0=none, 99=unknown |
| Did the patient ever attempt suicide? | 1=yes, 0=no, 99=unknown |
| Number of suicide attempts in life | Number of suicide attempts in life, 99=unknown |
| Current smoker (at time of enrollment) (note: former smoker is defined as completely abstinent for 6 months or more) | 1=yes, 0=not present, 99=unknown |
| Alcohol intake (at time of enrollment) | 0=never, 1=Monthly or less, 2=two to four times a month, 3=two to three times a week, 4=four or more times a week, 99=unknown |
| Drug abuse (at time of enrollment) | 1=yes, 0=not present, 99=unknown |
| List of diseases co-occurring in the patient (non-psychiatric) at time of enrollment | Other diseases co-occurring in the patient (free text, each disease separated by a semi-colon) |
| Does patient take any concurrent medications, including psychiatric medication, time of enrollment | 1=yes, 0=no, 99=unknown |
| List all concurrent medication, including psychiatric medication, time of enrollment | drug name for concurrent medications, including psychiatric medication |
| Was at least one of the patient's first degree relatives ever diagnosed with depression? | 1=yes, 0=no, 99=unknown |
| Study inclusion diagnosis (free text) | Psychiatric diagnosis at study inclusion in free text |
| Study inclusion DSM_IV | DSM-IV (Diagnostic and Statistical Manual of Mental Disorders, Fourth Edition) diagnosis code (numerical) |
| Diagnosis subcode_DSM_IV | DSM-IV diagnosis subcode (numerical) |
| Study inclusion ICD-9 code | ICD-9 diagnosis code (numerical) |
| Antidepressant drug 1 at begin of study | Generic drug name for antidepressant drug 1 |
| Antidepressant drug 1 dosage at begin of study | Dose of antidepressant drug 1 (mg daily) |
| Antidepressant drug 2 at begin of study | Generic drug name for antidepressant drug 2 |
| Antidepressant drug 2 dosage at begin of study | Dose of antidepressant drug 2 (mg daily) |
| Is patient receiving psychotherapy? (at baseline) | 1=yes, 0=no, 99=unknown |
| Is the patient presently at suicide risk? (at baseline) | 1=yes, 0=no, 99=unknown |
| **S1c. Phenotypic data** |  |
| Baseline-Was the patient evaluated with HRSD and what item version was used. | 0=HRSD not used, 17=HRSD with 17 questions used, 21=HRSD with 21 questions used, 24=HRSD with 24 questions used |
| Baseline-HRSD-Initial (Early) Insomnia | 0=absent, 1=mild, 2=moderate to severe and obvious |
| Baseline-HRSD-Middle Insomnia | 0=absent, 1=mild, 2=moderate to severe |
| Baseline-HRSD-Delayed (Late) Insomnia | 0=absent, 1=mild, 2=moderate to severe |
| Baseline-HRSD-Depressed Mood | 0=not depressed, 1=mild, 2=moderate, 3=moderately severe, 4=severe |
| Baseline-HRSD-Anxiety (Psychic) | 0=absent, 1=mild, 2=moderate, 3=moderately severe, 4=severe |
| Baseline-HRSD-Loss of Insight | 0=acknowledges being depressed and ill, 1=acknowledges being ill but not depressed, 2=denies being ill at all |
| Baseline-HRSD-Somatic Symptoms, Gastrointestinal (Appetite) | 0=normal appetite, 1=mild reduction in appetite and food intake, 2=moderate to severe reduction of appetite and food intake |
| Baseline-HRSD-Weight Loss | 0=no weight loss, 1=mild or probable weight loss associated with present illness, 2=moderate to severe weight loss, 3=not assessed |
| Baseline-HRSD-Anxiety (Somatic) | 0=absent, 1=mild, 2=moderate, 3=moderately severe, 4=severe |
| Baseline-HRSD-Hypochondriasis | 0=absent, 1=mild preoccupation with bodily functions and physical symptoms, 2=moderate concern with physical health, 3=moderately severe preoccupation with physical health, 4=severe |
| Baseline-HRSD-Guilt Feelings and Delusions | 0=absent, 1=mild self-reproach, 2=moderate, 3=moderately severe, 4=severe self-reproach |
| Baseline-HRSD-Suicide | 0=absent, 1=feels life is empty, 2=recurrent wishes or thoughts about death of self, 3=active suicidal thoughts, threats, gestures, 4=serious suicide attempt |
| Baseline-HRSD-Work and Activities (Interests) | 0=no disturbance, 1=mild, 2=moderate, 3=moderately severe, 4=severe |
| Baseline-HRSD-Somatic Symptoms, General | 0=normal, 1=mild, 2=moderate to severe |
| Baseline-HRSD-Retardation | 0=absent, 1=mild, 2=moderate, 3=moderately severe, 4=extremely severe |
| Baseline-HRSD-Agitation | 0=absent, 1=mild, 2=moderate, 3=moderately severe, 4=severe |
| Baseline-HRSD-Genital Symptoms (Libido, Menstrual Disturbances) | 0=absent, 1=mild, 2=moderate to severe |
| Baseline-HRSD-Diurnal Variation | 0=no variation, 1=worse in A.M., 2=worse in P.M. |
| Baseline-HRSD-Depersonalization and Derealization | 0=absent, 1=mild, 2=moderate, 3=severe, 4=incapacitating |
| Baseline-HRSD-Paranoid Symptoms | 0=none, 1=suspicious, 2=ideas of reference, 3=delusions of reference and persecution |
| Baseline-HRSD-Obsessional and compulsive Symptoms | 0=absent, 1=mild, 2=severe |
| ***Follow-up 1**** |  |
| Time period Baseline-Follow-up1 | Time period between baseline (study start) and follow-up1 (weeks) |
| Follow-up1-Clinical Global Impression | CGI: 1=Very Much, Improved, 2=Much Improved, 3=Minimally, Improved, 4=No Change, 5=Minimally, Worse, 6=Much Worse, 7=Very much worse, 99=unknown |
| Follow-up1-If you collected data on compliance, how was compliance to medication defined? | free text (e.g. blood draw, pill count, med taken under supervision) |
| Follow-up1-Did patient adhere to medication? | 1=yes, 0=no, 99=unknown |
| Follow-up1-If you collected data on side effects, did the patient experienced side effects? | 1=yes, 0=no, 99=unknown |
| Follow-up1-List of side effects patient experienced? | List side effects as free text separated by semicolon |
| Follow-up1-Did patient withdraw from study? | 1=yes, 0=no, 99=unknown |
| Follow-up1-Reason for withdrawal | 1=adverse effects, 2=lack of response, 3=lack of treatment adherence, 4=other reason, 99=unknown |
| Follow-up1-Is the patient presently at suicide risk? | 1=yes, 0=no, 99=unknown |
| Follow-up1-Dose Adjustment | Will you adjust the dose? 1=yes, 0=no, 99=unknown |
| Follow-up1-Dosage | New dosage (mg) |
| Follow-up1-No Dose Increased | Reason dose not raised 1=side effects, 2=dose is efficient, 3=other, 99=unknown |
| Follow-up1-Change of medication | Will the patient switch drugs? 1=yes, 0=no, 99=unknown |
| Follow-up1-Reason for change of medication | 1=side effects, 2=not efficient, 3=other, 99=unknown |
| Follow-up1-Number of days to medication change | Number of days to medication change |
| Follow-up1-New Antidepressant Drug (if antidepressant changed) | Generic drug name for changed to antidepressant drug follow-up1 |
| Follow-up1-New Antidepressant Drug Dosage (if antidepressant changed) | Dose drug (mg daily) |
| Follow-up1-Was the patient evaluated with HRSD and what item version was used. | 0=HRSD not used, 17=HRSD with 17 questions used, 21=HRSD with 21 questions used, 24=HRSD with 24 questions used |
| Follow-up1-HRSD-Initial (Early) Insomnia | 0=absent, 1=mild, 2=moderate to severe and obvious |
| Follow-up1-HRSD-Middle Insomnia | 0=absent, 1=mild, 2=moderate to severe |
| Follow-up1-HRSD-Delayed (Late) Insomnia | 0=absent, 1=mild, 2=moderate to severe |
| Follow-up1-HRSD-Depressed Mood | 0=not depressed, 1=mild, 2=moderate, 3=moderately severe, 4=severe |
| Follow-up1-HRSD-Anxiety (Psychic) | 0=absent, 1=mild, 2=moderate, 3=moderately severe, 4=severe |
| Follow-up1-HRSD-Loss of Insight | 0=acknowledges being depressed and ill, 1=acknowledges being ill but not depressed, 2=denies being ill at all |
| Follow-up1-HRSD-Somatic Symptoms, Gastrointestinal (Appetite) | 0=normal appetite, 1=mild reduction in appetite and food intake, 2=moderate to severe reduction of appetite and food intake |
| Follow-up1-HRSD-Weight Loss | 0=no weight loss, 1=mild or probable weight loss associated with present illness, 2=moderate to severe weight loss, 3=not assessed |
| Follow-up1-HRSD-Anxiety (Somatic) | 0=absent, 1=mild, 2=moderate, 3=moderately severe, 4=severe |
| Follow-up1-HRSD-Hypochondriasis | 0=absent, 1=mild preoccupation with bodily functions and physical symptoms, 2=moderate concern with physical health, 3=moderately severe preoccupation with physical health, 4=severe |
| Follow-up1-HRSD-Guilt Feelings and Delusions | 0=absent, 1=mild self-reproach, 2=moderate, 3=moderately severe, 4=severe self-reproach |
| Follow-up1-HRSD-Suicide | 0=absent, 1=feels life is empty, 2=recurrent wishes or thoughts about death of self, 3=active suicidal thoughts, threats, gestures, 4=serious suicide attempt |
| Follow-up1-HRSD-Work and Activities (Interests) | 0=no disturbance, 1=mild, 2=moderate, 3=moderately severe, 4=severe |
| Follow-up1-HRSD-Somatic Symptoms, General | 0=normal, 1=mild, 2=moderate to severe |
| Follow-up1-HRSD-Retardation | 0=absent, 1=mild, 2=moderate, 3=moderately severe, 4=extremely severe |
| Follow-up1-HRSD-Agitation | 0=absent, 1=mild, 2=moderate, 3=moderately severe, 4=severe |
| Follow-up1-HRSD-Genital Symptoms (Libido, Menstrual Disturbances) | 0=absent, 1=mild, 2=moderate to severe |
| Follow-up1-HRSD-Diurnal Variation | 0=no variation, 1=worse in A.M., 2=worse in P.M. |
| Follow-up1-HRSD-Depersonalization and Derealization | 0=absent, 1=mild, 2=moderate, 3=severe, 4=incapacitating |
| Follow-up1-HRSD-Paranoid Symptoms | 0=none, 1=suspicious, 2=ideas of reference, 3=delusions of reference and persecution |
| Follow-up1-HRSD-Obsessional and compulsive Symptoms | 0=absent, 1=mild, 2=severe |
| Did patient commit suicide during study? | Patient committed suicide; 1=yes, 0=no, 99=unknown |
| suicide date | How many weeks after baseline |

*Note: These data were collected for each available follow-up time

**Supplemental Table S3**: Top SNP association regions for observed and imputed SNPs in the ISPC sample, along with replication analysis using AMPS and STAR*D data.

|  |  |  |  |  |  |  |  |  |  |  | ISPC | | AMPS | | STAR*D | |
| --- | --- | --- | --- | --- | --- | --- | --- | --- | --- | --- | --- | --- | --- | --- | --- | --- |
| Outcome | SNP | CHR | BP | I/O | gene | MA | CA | MAF | C-Frq | A-Frq | beta/ OR | P | beta/ OR | P | beta/ OR | P |
| %change in depression score | rs56058016 | 1 | 20609464 | I | VWA5B1 | T | C | 0.01 | 0.04 | 0.00 | -1.47 | 1.13E-07 | NA | NA | NA | NA |
|  | rs4747621 | 10 | 28770717 | I | LOC220906/ WAC | C | A | 0.32 | 0.38 | 0.28 | -0.28 | 1.75E-07 | -0.07 | 0.31 | 0.02 | 0.66 |
|  | rs7041589 | 9 | 110965171 | I | LOC100128657 | G | C | 0.07 | 0.18 | 0.01 | -0.49 | 5.40E-07 | -0.03 | 0.71 | -0.05 | 0.25 |
|  | rs113243734 | 7 | 138697944 | I | ZC3HAV1L | C | T | 0.01 | 0.03 | 0.00 | 1.13 | 5.64E-07 | -0.15 | 0.42 | -0.03 | 0.74 |
|  | rs9328202 | 6 | 3887339 | O | RPS25P7 | A | C | 0.03 | 0.04 | 0.02 | 0.72 | 6.15E-07 | 0.28 | 0.16 | NA | NA |
|  | rs11989215 | 8 | 6395909 | O | ANGPT2/ MCPH1 | G | A | 0.36 | 0.36 | 0.36 | 0.24 | 7.46E-07 | -0.02 | 0.78 | NA | NA |
|  | rs16855294 | 2 | 169199695 | O | STK39 | A | C | 0.42 | 0.6 | 0.32 | -0.24 | 8.82E-07 | -0.03 | 0.63 | 0.03 | 0.44 |
|  | rs12355629 | 10 | 68504487 | I | CTNNA3 | C | T | 0.02 | 0.05 | 0.00 | -1.02 | 1.04E-06 | -0.19 | 0.41 | -0.13 | 0.34 |
|  | rs9520830 | 13 | 108935308 | I | TNFSF13B | C | A | 0.04 | 0.08 | 0.02 | 1.06 | 1.24E-06 | 0.34 | 0.07 | NA | NA |
|  | rs73198127 | 8 | 17442885 | I | PDGFRL | A | G | 0.09 | 0.25 | 0.01 | -0.41 | 1.68E-06 | -0.03 | 0.72 | -0.08 | 0.07 |
| response | rs3782401 | 12 | 54911983 | I | NCKAP1L | A | G | 0.42 | 0.17 | 0.55 | 2.13 | 7.03E-07 | 1.02 | 0.90 | 0.96 | 0.80 |
|  | rs1348903 | 2 | 167386717 | I | SCN7A | T | C | 0.45 | 0.62 | 0.36 | 1.67 | 2.85E-06 | 1.17 | 0.25 | 1.00 | 0.97 |
|  | rs4747621 | 10 | 28770717 | I | LOC220906/ WAC | C | A | 0.32 | 0.38 | 0.28 | 1.72 | 2.88E-06 | 1.05 | 0.73 | 0.95 | 0.56 |
|  | rs17236056 | 15 | 33720854 | I | RYR3 | A | G | 0.12 | 0.18 | 0.09 | 0.35 | 3.52E-06 | 1.21 | 0.27 | NA | NA |
|  | rs41273882 | 6 | 53517228 | I | KLHL31 | C | A | 0.08 | 0.08 | 0.08 | 0.41 | 5.06E-06 | 0.91 | 0.71 | 1.13 | 0.39 |
|  | rs8088956 | 18 | 4973174 | I | PPIAP14 | A | G | 0.47 | 0.52 | 0.44 | 1.59 | 6.19E-06 | 1.09 | 0.53 | 0.95 | 0.56 |
|  | rs11989215 | 8 | 6395909 | O | ANGPT2/ MCPH1 | G | A | 0.36 | 0.36 | 0.36 | 0.62 | 6.70E-06 | 1.18 | 0.27 | NA | NA |
|  | rs114548043 | 6 | 32963844 | I | HLA-DOA | A | T | 0.05 | 0.02 | 0.07 | 0.27 | 1.00E-05 | 0.68 | 0.36 | NA | NA |
|  | rs12024635 | 1 | 65568853 | I | MRPS21P1 | A | T | 0.14 | 0.08 | 0.16 | 0.51 | 1.23E-05 | 0.96 | 0.90 | 0.94 | 0.63 |
|  | rs11120788 | 1 | 6992858 | I | CAMTA1 | A | G | 0.24 | 0.34 | 0.19 | 1.71 | 1.24E-05 | 0.91 | 0.51 | 0.98 | 0.81 |

I/O = indicator of whether the SNP was observed (O) or imputed (I); CA = common allele; MA = minor allele; MAF = minor allele frequency; C-Frq = Frequency of MA in subset; A-Frq = Frequency of MA in Asian subset; Beta/OR = regression parameter estimates (beta) for SNP effect on quantitative trait outcome or odds ratio (OR) estimate for SNP effect on binary outcome

**Supplemental Table S4:** Replication of top results from recent large-sample pharmacogenomics GWAS of antidepressant response[^23^](#_ENREF_23)^,^ [^24^](#_ENREF_24)

| SNP | gene | chr | Prior evidence | | ISPC %change | | ISPC response | | Meta-analysis* %change | | Meta-analysis* response | |
| --- | --- | --- | --- | --- | --- | --- | --- | --- | --- | --- | --- | --- |
|  |  |  | Associated Outcome | P | beta | P | OR | P | beta | P | OR | P |
| rs17651119 | MYO10 | 5 | 12 week %improvement, entire sample [^24^](#_ENREF_24) | 1.78E-08 | 0.060 | 0.39 | 0.89 | 0.46 | -0.060 | 0.44 | 0.91 | 0.53 |
| rs2546057 | KCTD15 | 19 | 12 week %improvement, entire sample [^24^](#_ENREF_24) | 2.56E-07 | -0.110 | 0.076 | 1.33 | **0.027** | -0.030 | 0.53 | 1.06 | 0.53 |
| rs12410462 | -- | 1 | 12 week %improvement, entire sample [^24^](#_ENREF_24) | 4.20E-07 | 0.040 | 0.60 | 0.93 | 0.64 | 0.000 | 0.93 | 1.01 | 0.93 |
| rs7174755 | ITGA11 | 15 | 2 week % improvement, entire sample [^24^](#_ENREF_24) | 9.12E-07 | 0.090 | 0.24 | 0.89 | 0.47 | -0.130 | **0.010** | 0.79 | **0.025** |
| rs10065906 |  | 5 | 2 week partial response, entire sample [^24^](#_ENREF_24) | 5.29E-08 | 0.020 | 0.74 | 0.96 | 0.71 | -0.070 | 0.079 | 0.87 | 0.12 |
| rs17538444 | ENOX1 | 13 | 12 week %improvement, SSRI subset [^24^](#_ENREF_24) | 4.17E-07 | -0.160 | 0.33 | 1.33 | 0.42 | 0.200 | 0.066 | 1.52 | 0.071 |
| rs12054895 | -- | 5 | 2 week % improvement, SSRI subset [^24^](#_ENREF_24) | 2.65E-08 | -0.040 | 0.44 | 1.11 | 0.33 | 0.010 | 0.72 | 1.03 | 0.71 |
| rs4585146 | FGF12 | 3 | 2 week % improvement, SSRI subset [^24^](#_ENREF_24) | 3.00E-07 | 0.030 | 0.59 | 0.99 | 0.91 | 0.020 | 0.59 | 0.97 | 0.73 |
| rs10818702 | ORIJ2 | 9 | Response to any antidepressant [^23^](#_ENREF_23) | 2.19E-06 | -0.070 | 0.21 | 0.99 | 0.95 | -0.010 | 0.67 | 0.97 | 0.71 |
| rs11624702 | MDGA2 | 14 | Response to any antidepressant [^23^](#_ENREF_23) | 4.08E-06 | 0.040 | 0.37 | 0.83 | 0.061 | 0.030 | 0.19 | 0.89 | **0.029** |
| rs7708972 | ADAMTS6 | 5 | Response to serotonergic antidepressant [^23^](#_ENREF_23) | 2.49E-06 | -0.010 | 0.91 | 0.93 | 0.47 | 0.010 | 0.75 | 0.98 | 0.71 |
| rs1493451 | ADAMTS6 | 5 | Response to serotonergic antidepressant [^23^](#_ENREF_23) | 2.35E-06 | -0.040 | 0.37 | 1.05 | 0.64 | -0.010 | 0.79 | 1.00 | 0.97 |
| rs10515893 | -- | 5 | Response to serotonergic antidepressant [^23^](#_ENREF_23) | 1.37E-06 | 0.050 | 0.45 | 0.86 | 0.26 | 0.010 | 0.75 | 1.00 | 1.0 |
| rs10783282 | ADCY6 | 12 | Response to serotonergic antidepressant [^23^](#_ENREF_23) | 1.16E-06 | 0.020 | 0.66 | 0.92 | 0.49 | 0.010 | 0.80 | 1.05 | 0.40 |

*Note that for SNPs previously reported by Tansey et al ^[23](#_ENREF_23)^, we tested replication using our ISPC sample, and our combined ISPC+AMPS+STAR*D meta-analysis. However, for SNPs previously reported by Uher et al. ^[24](#_ENREF_24)^, we tested for replication using our ISPC sample, and our combined ISPC+AMPS analysis (without STAR*D) because the analysis of Uher et al. included STAR*D samples.**Supplemental Figure S1**: Structure analysis plot for ISPC subjects.


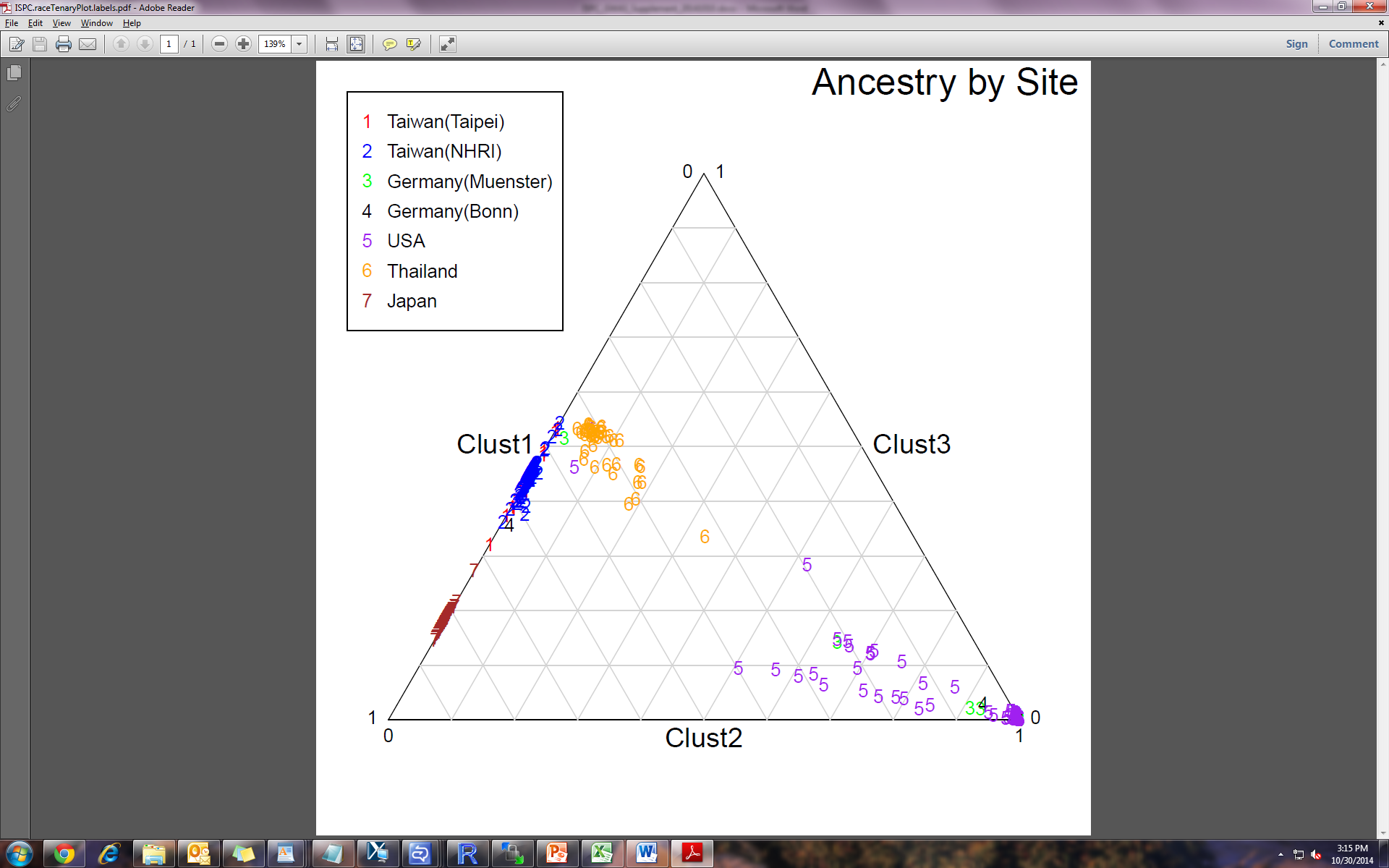


**Supplemental Figure S2**: QQ plots for primary analyses of ISPC data including observed and imputed SNPs (A) %ΔHRSD (B) Response


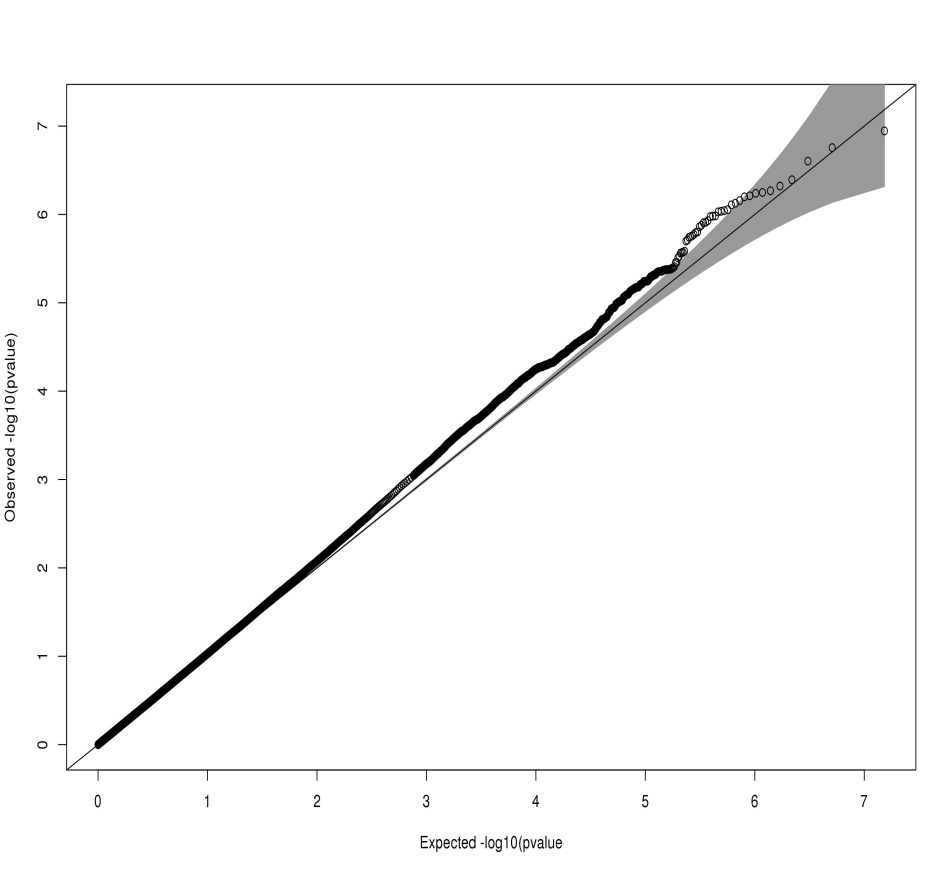

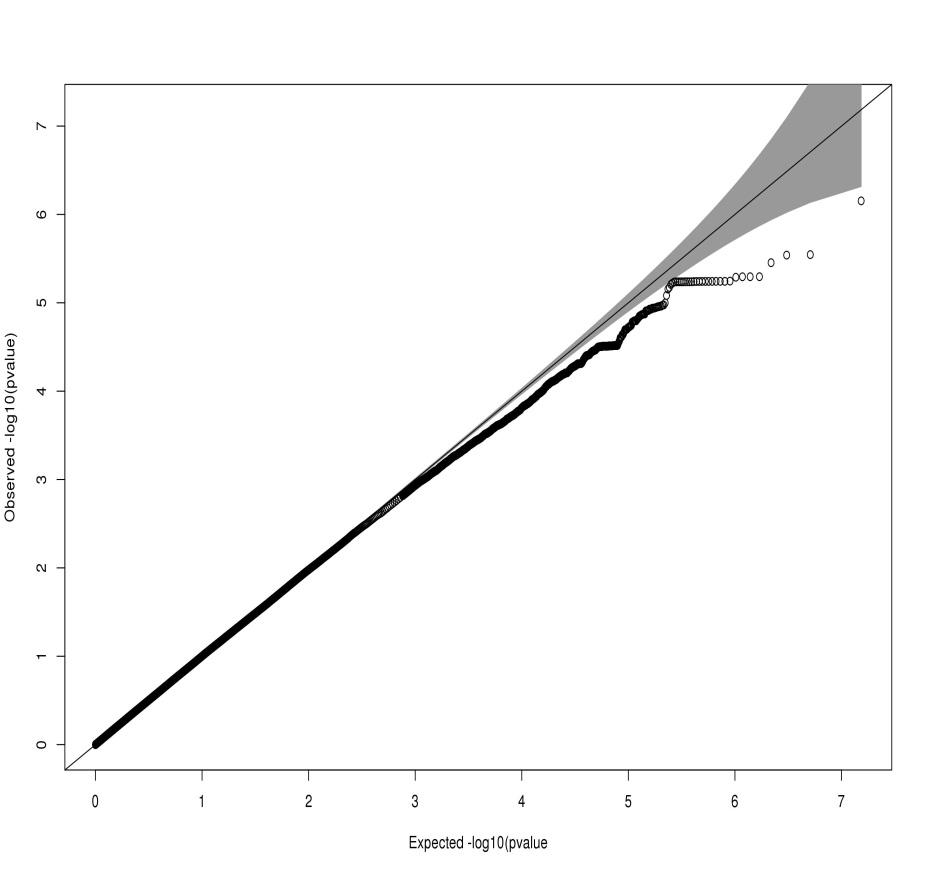


B

A

**Supplemental Figure S3**: QQ plots for meta-analyses of ISPC, AMPS and STAR*D data (A) %Δ depression score (B) Response


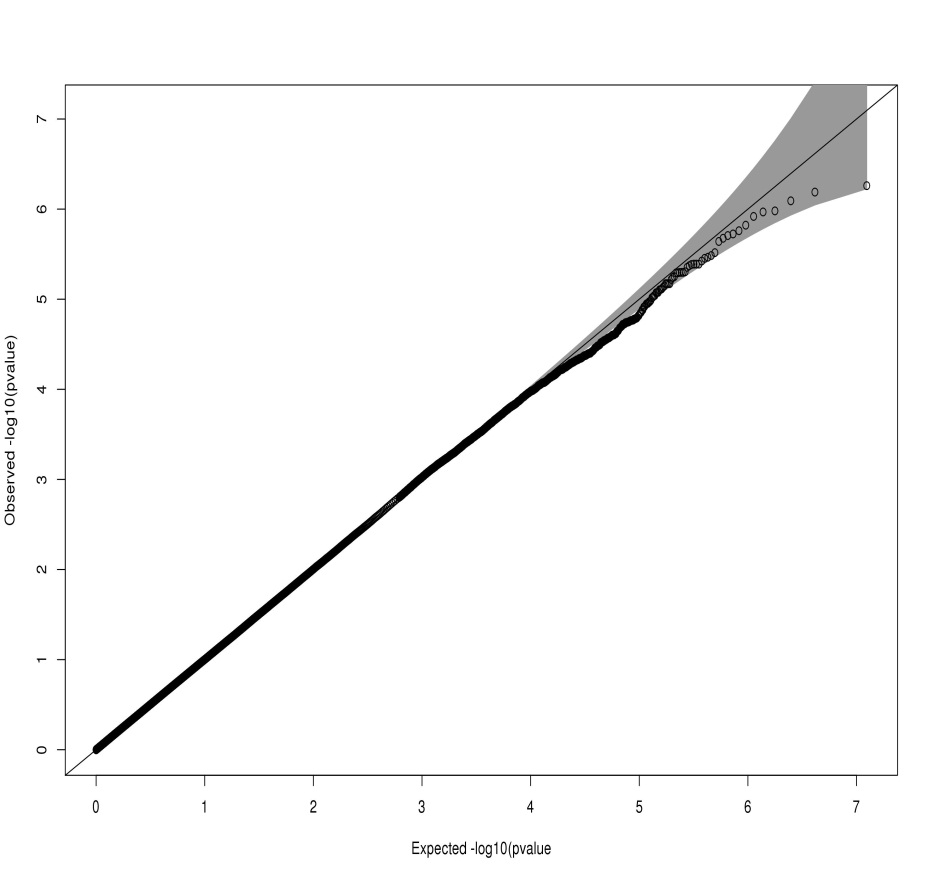

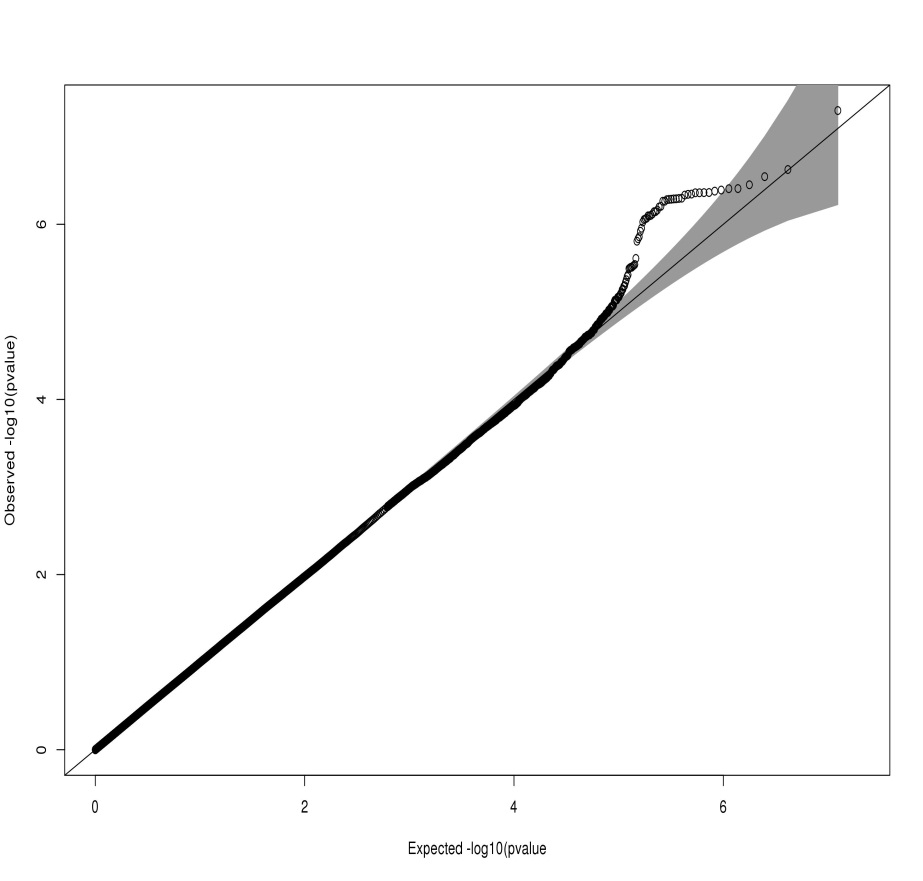


A

B

**Supplemental Figure S4**: LocusZoom plot of results from meta-analysis of “response” in the *HPRTP4* region


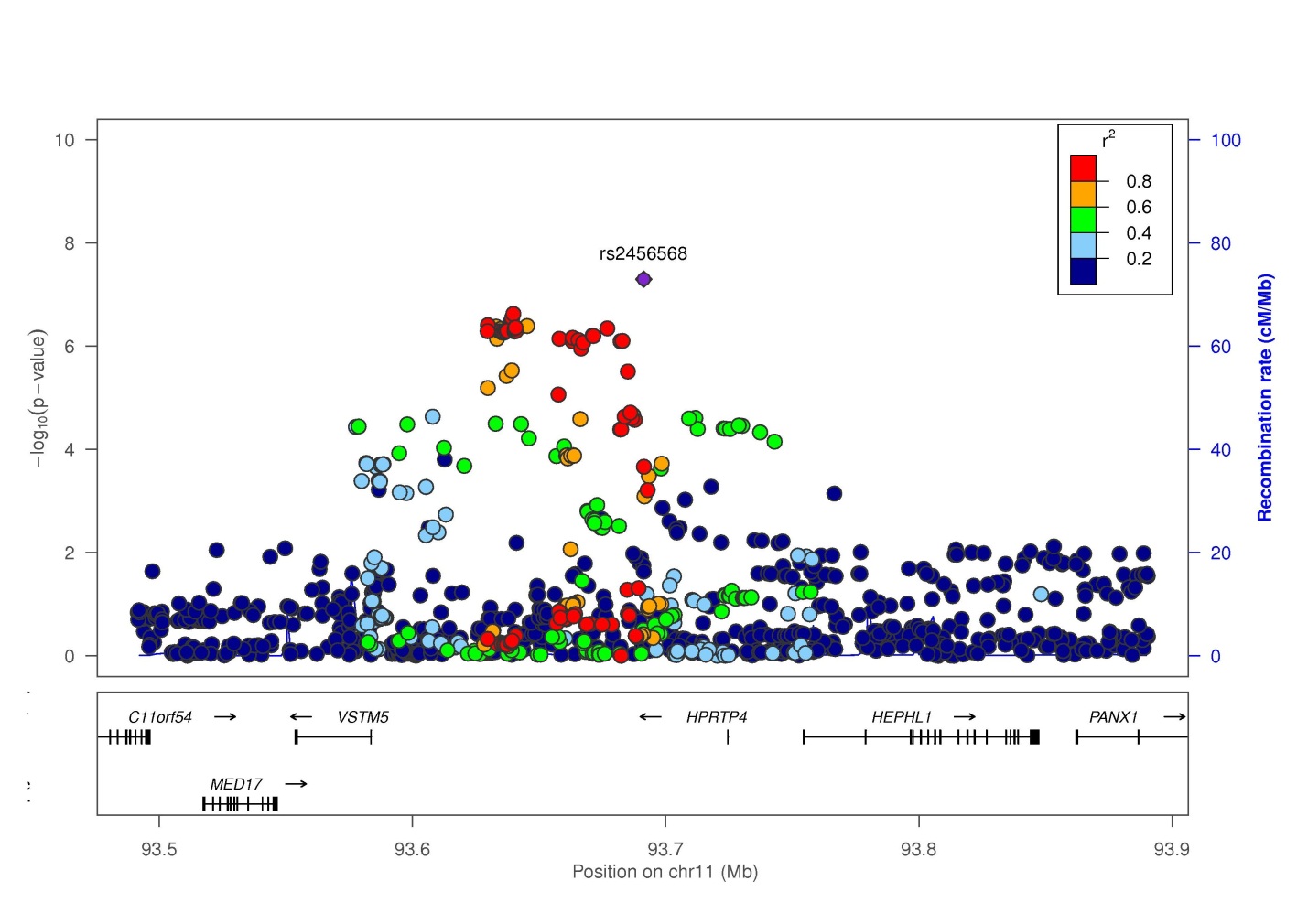


Top 5 SNPs in Figure S4

| SNP | CHR | BP | I/O* | OR | pvalue | gene |
| --- | --- | --- | --- | --- | --- | --- |
| rs2456568 | 11 | 93691332 | I | 1.36 | 5.03E-08 | HPRTP4 |
| rs1506656 | 11 | 93639813 | I | 1.34 | 2.37E-07 | VSTM5 |
| rs1395386 | 11 | 93639331 | I | 1.33 | 2.86E-07 | VSTM5 |
| rs11020585 | 11 | 93638493 | I | 1.33 | 3.54E-07 | VSTM5 |
| rs11020586 | 11 | 93638547 | I | 1.33 | 3.91E-07 | VSTM5 |
| rs1108912 | 11 | 93629756 | I | 1.33 | 3.92E-07 | VSTM5 |
| rs10765643 | 11 | 93645269 | I | 1.38 | 4.06E-07 | VSTM5 |

*I/O indicator of whether the SNP was observed (O) or imputed (I). IO means the SNP was observed in at least one of the datasets contributing to the meta-analysis and imputed in at least one dataset.

**Supplemental Figure S5**: LocusZoom plot of results from meta-analysis of “response” in the *NRG1* region


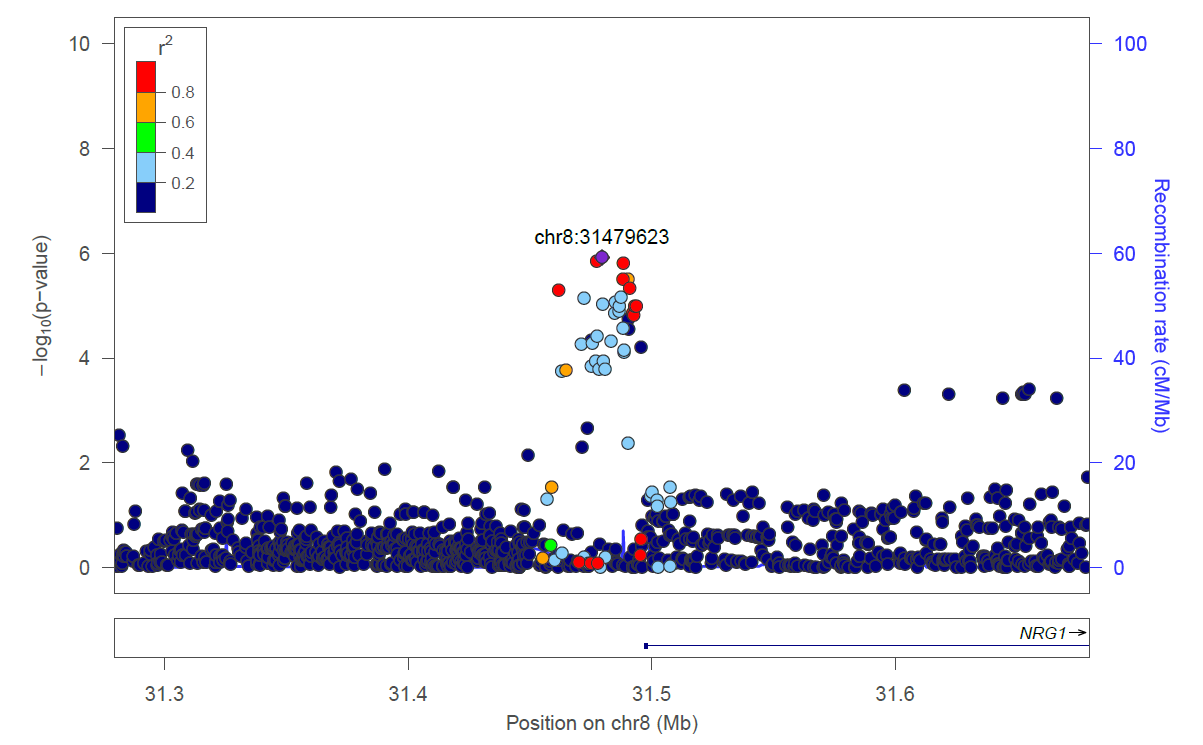


Top 5 SNPs in Figure S5

| SNP | CHR | BP | I/O* | OR | pvalue | gene |
| --- | --- | --- | --- | --- | --- | --- |
| rs10954808 | 8 | 31479623 | I | 0.73 | 1.20E-06 | NRG1 |
| rs12680816 | 8 | 31477431 | I | 0.73 | 1.39E-06 | NRG1 |
| rs12546600 | 8 | 31488328 | I | 0.73 | 1.56E-06 | NRG1 |
| rs12546840 | 8 | 31490319 | I | 0.72 | 3.09E-06 | NRG1 |
| rs12541452 | 8 | 31488275 | IO | 1.36 | 3.15E-06 | NRG1 |

*I/O indicator of whether the SNP was observed (O) or imputed (I). IO means the SNP was observed in at least one of the datasets contributing to the meta-analysis and imputed in at least one dataset.

REFERENCES

1. Liou YJ, Chen CH, Cheng CY, Chen SY, Chen TJ, Yu YW *et al.* Convergent evidence from mouse and human studies suggests the involvement of zinc finger protein 326 gene in antidepressant treatment response. . *PLoS One* 2012; **7**(5)**:** e32984.

2. Tsai SJ, Liou YJ, Hong CJ, Yu YW, Chen TJ. Glycogen synthase kinase-3beta gene is associated with antidepressant treatment response in Chinese major depressive disorder. *Pharmacogenomics J* 2008; **8**(6)**:** 384-390.

3. Yu YW, Chen TJ, Hong CJ, Chen HM, Tsai SJ. Association study of the interleukin-1 beta (C-511T) genetic polymorphism with major depressive disorder, associated symptomatology, and antidepressant response. *Neuropsychopharmacology* 2003; **28**(6)**:** 1182-1185.

4. Kuo HW, Liu SC, Tsou HH, Liu SW, Lin KM, Lu SC *et al.* CYP1A2 genetic polymorphisms are associated with early antidepressant escitalopram metabolism and adverse reactions. *Pharmacogenomics* 2013; **14**(10)**:** 11.

5. Lin HL, Hsu YT, Liu CY, Hsiao CF, Hsiao MC, Liu YL *et al.* Comparison of escitalopram and paroxetine in the treatment of major depressive disorder. *Int Clin Psychopharmacol* 2013; **28**(6)**:** 7.

6. Lin KM, Chiu YF, Tsai IJ, Chen CH, Shen WW, Liu SC *et al.* ABCB1 gene polymorphisms are associated with the severity of major depressive disorder and its response to escitalopram treatment. *Pharmacogenet Genomics* 2011; **21**(4)**:** 163-170.

7. Baffa A, Hohoff C, Baune BT, Müller-Tidow C, Tidow N, Freitag C *et al.* Norepinephrine and serotonin transporter genes: impact on treatment response in depression. *Neuropsychobiology* 2010; **62:** 11.

8. Baune BT, Hohoff C, Berger K, Neumann A, Mortensen S, Roehrs T *et al.* Association of the COMT val158met variant with antidepressant treatment response in major depression. *Neuropsychopharmacology* 2008; **33:** 9.

9. Domschke K, Hohoff C, Mortensen L, Roehrs T, Deckert J, Arolt V *et al.* Monoamine oxidase A variant influences antidepressant treatment response in female patients with Major Depression. *Prog Neuropsychopharmacol Biol Psychiatry* 2008; **32:** 5.

10. Kirchheiner J, Lorch R, Lebedeva E, Seeringer A, Roots I, Sasse J *et al.* Genetic variants in FKBP5 affecting response to antidepressant drug treatment. *Pharmacogenomics* 2008; **9**(7)**:** 6.

11. Kirchheiner J, Nickchen K, Sasse J, Bauer M, Roots I, Brockmöller J. A 40-basepair VNTR polymorphism in the dopamine transporter (DAT1) gene and the rapid response to antidepressant treatment. *Pharmacogenomics J* 2007; **7**(1)**:** 48-55.

12. Tzvetkov MV, Brockmöller J, Roots I, Kirchheiner J. Common genetic variations in human brain-specific tryptophan hydroxylase-2 and response to antidepressant treatment. *Pharmacogenet Genomics* 2007; **18**(6)**:** 495-506.

13. Ji Y, Biernacka JM, Hebbring S, Chai Y, Jenkins GD, Batzler A *et al.* Pharmacogenomics of selective serotonin reuptake inhibitor treatment for major depressive disorder: genome-wide associations and functional genomics. *Pharmacogenomics J* 2013; **13**(5)**:** 456-463.

14. Mrazek DA, Biernacka JM, McAlpine DE, Benitez J, Karpyak VM, Williams MD *et al.* Treatment outcomes of depression: The pharmacogenomic research network antidepressant medication pharmacogenomic study. *Journal of Clinical Psychopharmacology* 2014; **34**(3)**:** 313-317.

15. Kato M, Fukuda T, Wakeno M, Fukuda K, Okugawa G, Ikenaga Y *et al.* Effects of the serotonin type 2A, 3A and 3B receptor and the serotonin transporter genes on paroxetine and fluvoxamine efficacy and adverse drug reactions in depressed Japanese patients. *Neuropsychobiology* 2006; **53**(4)**:** 186-195.

16. Kato M, Ikenaga Y, Wakeno M, Okugawa G, Nobuhara K, Fukuda T *et al.* Controlled clinical comparison of paroxetine and fluvoxamine considering the serotonin transporter promoter polymorphism. *Int Clin Psychopharmacol* 2005; **20**(3)**:** 151-156.

17. Kato M, Okugawa G, Wakeno M, Takekita Y, Nonen S, Tetsuo S *et al.* Effect of basic fibroblast growth factor (FGF2) gene polymorphisms on SSRIs treatment response and side effects. *Eur Neuropsychopharmacol* 2009; **19**(10)**:** 718-725.

18. Purcell S, Neale B, Todd-Brown K, Thomas L, Ferreira MA, Bender D *et al.* PLINK: a tool set for whole-genome association and population-based linkage analyses. *Am J Hum Genet* 2007; **81**(3)**:** 559-575.

19. Pritchard JK, Stephens M, Donnelly P. Inference of population structure using multilocus genotype data. *Genetics* 2000; **155**(2)**:** 945-959.

20. Illi A, Setala-Soikkeli E, Viikki M, Poutanen O, Huhtala H, Mononen N *et al.* 5-HTR1A, 5-HTR2A, 5-HTR6, TPH1 and TPH2 polymorphisms and major depression. *Neuroreport* 2009; **20**(12)**:** 1125-1128.

21. Ji Y, Biernacka JM, Hebbring S, Chai Y, Jenkins GD, Batzler A *et al.* Pharmacogenomics of selective serotonin reuptake inhibitor treatment for major depressive disorder: genome-wide associations and functional genomics. *Pharmacogenomics J* 2013; **13**(5)**:** 456-463.

22. Mrazek DA, Biernacka JM, McAlpine DE, Benitez J, Karpyak VM, Williams MD *et al.* Treatment outcomes of depression: the pharmacogenomic research network antidepressant medication pharmacogenomic study. *J Clin Psychopharmacol* 2014; **34**(3)**:** 313-317.

23. Tansey KE, Guipponi M, Perroud N, Bondolfi G, Domenici E, Evans D *et al.* Genetic predictors of response to serotonergic and noradrenergic antidepressants in major depressive disorder: a genome-wide analysis of individual-level data and a meta-analysis. *PLoS Med* 2012; **9**(10)**:** e1001326.

24. Uher R, Investigators G, Investigators M, Investigators SD. Common genetic variation and antidepressant efficacy in major depressive disorder: a meta-analysis of three genome-wide pharmacogenetic studies. *Am J Psychiatry* 2013; **170**(2)**:** 207-217.
