# Supplementary material for: The International SSRI Pharmacogenomics Consortium (ISPC): a genome-wide association study of antidepressant treatment response
Source: Transl Psychiatry. 2016 Nov 1;6(11):e937–. doi: 10.1038/tp.2016.187 (PMC5314112; doi:10.1038/tp.2016.187)
Supplement: Supplementary file 1 [file tp2016187x1.docx]

**Supplemental Table S1**: ISPC Participating Sites and Study Description

| Study | Site | PI | Medication(s) | Study Duration | References |
| --- | --- | --- | --- | --- | --- |
| 1 | Taipei Veterans General Hospital, Taipei, Taiwan | Shih-Jen Tsai  Chen-Jee Hong  Ying-Jay Liou  Younger WY Yu  Tai-Jui Chen | fluoxetine, citalopram | 8 weeks | ^1-3^ |
| 2 | Center for Neuropsychiatric Research, National Health Research Institutes, Miaoli County, Taiwan | Chia-Hui Chen  Yu-Li Liu | escitalopram, paroxetine | 8 weeks | ^4-6^ |
| 3 | Tampere University Hospital, Finland | Ari Illi Esa Leinonen Olli Kampman | Fluoxetine Paroxetine Citalopram | 6 weeks | ^20^ |
| 4 | Muenster University Hospital, Germany | Volker Arolt  Bernhard Baune,  Katharina Domschke | escitalopram, paroxetine and sertraline | 6 weeks | ^7-9^ |
| 5 | University of Göttingen, Germany and Federal Institute for Drugs and Medical Devices, Bonn, Germany | Jürgen Brockmöller and Julia Stingl (former name Kirchheiner) | citalopram, escitalopram, paroxetine, sertraline, venlafaxine | 3 weeks observation in hospital | ^10-12^ |
| 6 | Mayo Clinic, USA | David A. Mrazek  Joanna Biernacka  Richard Weinshilboum | citalopram, escitalopram | 8 weeks | ^21^^,^ ^22^ |
| 7 | Rajanukul Institute, Thailand | Verayuth Praphanphoj | fluoxetine | 8 weeks |  |
| 8 | Kansai Medical University, Japan | Masaki Kato  Masataka Wakeno | paroxetine, fluvoxamine | 6 weeks | ^15-17^ |

**Supplemental Table S2:** Data Collected from Participating Sites

| **Demographic Data** | |
| --- | --- |
| Gender | Male or female |
| Race | Self-reported information and racial categories used as defined by the U.S. Office of Management and Budget |
| Ethnicity | Self-reported information and racial categories used as defined by the U.S. Office of Management and Budget |
| Age | Age at time of consent in years |
| **Baseline Data** | |
| Height | Height in centimeter |
| Weight | Weight in kilogram |
| What is patient's current employment status? | Unemployed, 2=employed, 3=retired, 99=unknown |
| Is patient married or in a long-term relationship? | 1=yes, 0=no, 2=widow/widower, 99=unknown |
| Does patient live with spouse or life partner? | 1=yes, 0=no, 99=unknown |
| Age at onset of first episode of depression | years, 99=unknown |
| Number of depressive episodes until time of study enrollment | Number, 99=unknown |
| Number of manic episodes until time of study enrollment | Number, 0=none, 99=unknown |
| Did the patient ever attempt suicide? | 1=yes, 0=no, 99=unknown |
| Number of suicide attempts in life | Number of suicide attempts in life, 99=unknown |
| Current smoker (at time of enrollment) (note: former smoker is defined as completely abstinent for 6 months or more) | 1=yes, 0=not present, 99=unknown |
| Alcohol intake (at time of enrollment) | 0=never, 1=Monthly or less, 2=two to four times a month, 3=two to three times a week, 4=four or more times a week, 99=unknown |
| Drug abuse (at time of enrollment) | 1=yes, 0=not present, 99=unknown |
| List of diseases co-occurring in the patient (non-psychiatric) at time of enrollment | Other diseases co-occurring in the patient (free text, each disease separated by a semi-colon) |
| Does patient take any concurrent medications, including psychiatric medication, time of enrollment | 1=yes, 0=no, 99=unknown |
| List all concurrent medication, including psychiatric medication, time of enrollment | drug name for concurrent medications, including psychiatric medication |
| Was at least one of the patient's first degree relatives ever diagnosed with depression? | 1=yes, 0=no, 99=unknown |
| Study inclusion diagnosis (free text) | Psychiatric diagnosis at study inclusion in free text |
| Study inclusion DSM_IV | DSM-IV (Diagnostic and Statistical Manual of Mental Disorders, Fourth Edition) diagnosis code (numerical) |
| Diagnosis subcode_DSM_IV | DSM-IV diagnosis subcode (numerical) |
| Study inclusion ICD-9 code | ICD-9 diagnosis code (numerical) |
| Antidepressant drug 1 at begin of study | Generic drug name for antidepressant drug 1 |
| Antidepressant drug 1 dosage at begin of study | Dose of antidepressant drug 1 (mg daily) |
| Antidepressant drug 2 at begin of study | Generic drug name for antidepressant drug 2 |
| Antidepressant drug 2 dosage at begin of study | Dose of antidepressant drug 2 (mg daily) |
| Is patient receiving psychotherapy? (at baseline) | 1=yes, 0=no, 99=unknown |
| Is the patient presently at suicide risk? (at baseline) | 1=yes, 0=no, 99=unknown |
| **S1c. Phenotypic data** |  |
| Baseline-Was the patient evaluated with HRSD and what item version was used. | 0=HRSD not used, 17=HRSD with 17 questions used, 21=HRSD with 21 questions used, 24=HRSD with 24 questions used |
| Baseline-HRSD-Initial (Early) Insomnia | 0=absent, 1=mild, 2=moderate to severe and obvious |
| Baseline-HRSD-Middle Insomnia | 0=absent, 1=mild, 2=moderate to severe |
| Baseline-HRSD-Delayed (Late) Insomnia | 0=absent, 1=mild, 2=moderate to severe |
| Baseline-HRSD-Depressed Mood | 0=not depressed, 1=mild, 2=moderate, 3=moderately severe, 4=severe |
| Baseline-HRSD-Anxiety (Psychic) | 0=absent, 1=mild, 2=moderate, 3=moderately severe, 4=severe |
| Baseline-HRSD-Loss of Insight | 0=acknowledges being depressed and ill, 1=acknowledges being ill but not depressed, 2=denies being ill at all |
| Baseline-HRSD-Somatic Symptoms, Gastrointestinal (Appetite) | 0=normal appetite, 1=mild reduction in appetite and food intake, 2=moderate to severe reduction of appetite and food intake |
| Baseline-HRSD-Weight Loss | 0=no weight loss, 1=mild or probable weight loss associated with present illness, 2=moderate to severe weight loss, 3=not assessed |
| Baseline-HRSD-Anxiety (Somatic) | 0=absent, 1=mild, 2=moderate, 3=moderately severe, 4=severe |
| Baseline-HRSD-Hypochondriasis | 0=absent, 1=mild preoccupation with bodily functions and physical symptoms, 2=moderate concern with physical health, 3=moderately severe preoccupation with physical health, 4=severe |
| Baseline-HRSD-Guilt Feelings and Delusions | 0=absent, 1=mild self-reproach, 2=moderate, 3=moderately severe, 4=severe self-reproach |
| Baseline-HRSD-Suicide | 0=absent, 1=feels life is empty, 2=recurrent wishes or thoughts about death of self, 3=active suicidal thoughts, threats, gestures, 4=serious suicide attempt |
| Baseline-HRSD-Work and Activities (Interests) | 0=no disturbance, 1=mild, 2=moderate, 3=moderately severe, 4=severe |
| Baseline-HRSD-Somatic Symptoms, General | 0=normal, 1=mild, 2=moderate to severe |
| Baseline-HRSD-Retardation | 0=absent, 1=mild, 2=moderate, 3=moderately severe, 4=extremely severe |
| Baseline-HRSD-Agitation | 0=absent, 1=mild, 2=moderate, 3=moderately severe, 4=severe |
| Baseline-HRSD-Genital Symptoms (Libido, Menstrual Disturbances) | 0=absent, 1=mild, 2=moderate to severe |
| Baseline-HRSD-Diurnal Variation | 0=no variation, 1=worse in A.M., 2=worse in P.M. |
| Baseline-HRSD-Depersonalization and Derealization | 0=absent, 1=mild, 2=moderate, 3=severe, 4=incapacitating |
| Baseline-HRSD-Paranoid Symptoms | 0=none, 1=suspicious, 2=ideas of reference, 3=delusions of reference and persecution |
| Baseline-HRSD-Obsessional and compulsive Symptoms | 0=absent, 1=mild, 2=severe |
| ***Follow-up 1**** |  |
| Time period Baseline-Follow-up1 | Time period between baseline (study start) and follow-up1 (weeks) |
| Follow-up1-Clinical Global Impression | CGI: 1=Very Much, Improved, 2=Much Improved, 3=Minimally, Improved, 4=No Change, 5=Minimally, Worse, 6=Much Worse, 7=Very much worse, 99=unknown |
| Follow-up1-If you collected data on compliance, how was compliance to medication defined? | free text (e.g. blood draw, pill count, med taken under supervision) |
| Follow-up1-Did patient adhere to medication? | 1=yes, 0=no, 99=unknown |
| Follow-up1-If you collected data on side effects, did the patient experienced side effects? | 1=yes, 0=no, 99=unknown |
| Follow-up1-List of side effects patient experienced? | List side effects as free text separated by semicolon |
| Follow-up1-Did patient withdraw from study? | 1=yes, 0=no, 99=unknown |
| Follow-up1-Reason for withdrawal | 1=adverse effects, 2=lack of response, 3=lack of treatment adherence, 4=other reason, 99=unknown |
| Follow-up1-Is the patient presently at suicide risk? | 1=yes, 0=no, 99=unknown |
| Follow-up1-Dose Adjustment | Will you adjust the dose? 1=yes, 0=no, 99=unknown |
| Follow-up1-Dosage | New dosage (mg) |
| Follow-up1-No Dose Increased | Reason dose not raised 1=side effects, 2=dose is efficient, 3=other, 99=unknown |
| Follow-up1-Change of medication | Will the patient switch drugs? 1=yes, 0=no, 99=unknown |
| Follow-up1-Reason for change of medication | 1=side effects, 2=not efficient, 3=other, 99=unknown |
| Follow-up1-Number of days to medication change | Number of days to medication change |
| Follow-up1-New Antidepressant Drug (if antidepressant changed) | Generic drug name for changed to antidepressant drug follow-up1 |
| Follow-up1-New Antidepressant Drug Dosage (if antidepressant changed) | Dose drug (mg daily) |
| Follow-up1-Was the patient evaluated with HRSD and what item version was used. | 0=HRSD not used, 17=HRSD with 17 questions used, 21=HRSD with 21 questions used, 24=HRSD with 24 questions used |
| Follow-up1-HRSD-Initial (Early) Insomnia | 0=absent, 1=mild, 2=moderate to severe and obvious |
| Follow-up1-HRSD-Middle Insomnia | 0=absent, 1=mild, 2=moderate to severe |
| Follow-up1-HRSD-Delayed (Late) Insomnia | 0=absent, 1=mild, 2=moderate to severe |
| Follow-up1-HRSD-Depressed Mood | 0=not depressed, 1=mild, 2=moderate, 3=moderately severe, 4=severe |
| Follow-up1-HRSD-Anxiety (Psychic) | 0=absent, 1=mild, 2=moderate, 3=moderately severe, 4=severe |
| Follow-up1-HRSD-Loss of Insight | 0=acknowledges being depressed and ill, 1=acknowledges being ill but not depressed, 2=denies being ill at all |
| Follow-up1-HRSD-Somatic Symptoms, Gastrointestinal (Appetite) | 0=normal appetite, 1=mild reduction in appetite and food intake, 2=moderate to severe reduction of appetite and food intake |
| Follow-up1-HRSD-Weight Loss | 0=no weight loss, 1=mild or probable weight loss associated with present illness, 2=moderate to severe weight loss, 3=not assessed |
| Follow-up1-HRSD-Anxiety (Somatic) | 0=absent, 1=mild, 2=moderate, 3=moderately severe, 4=severe |
| Follow-up1-HRSD-Hypochondriasis | 0=absent, 1=mild preoccupation with bodily functions and physical symptoms, 2=moderate concern with physical health, 3=moderately severe preoccupation with physical health, 4=severe |
| Follow-up1-HRSD-Guilt Feelings and Delusions | 0=absent, 1=mild self-reproach, 2=moderate, 3=moderately severe, 4=severe self-reproach |
| Follow-up1-HRSD-Suicide | 0=absent, 1=feels life is empty, 2=recurrent wishes or thoughts about death of self, 3=active suicidal thoughts, threats, gestures, 4=serious suicide attempt |
| Follow-up1-HRSD-Work and Activities (Interests) | 0=no disturbance, 1=mild, 2=moderate, 3=moderately severe, 4=severe |
| Follow-up1-HRSD-Somatic Symptoms, General | 0=normal, 1=mild, 2=moderate to severe |
| Follow-up1-HRSD-Retardation | 0=absent, 1=mild, 2=moderate, 3=moderately severe, 4=extremely severe |
| Follow-up1-HRSD-Agitation | 0=absent, 1=mild, 2=moderate, 3=moderately severe, 4=severe |
| Follow-up1-HRSD-Genital Symptoms (Libido, Menstrual Disturbances) | 0=absent, 1=mild, 2=moderate to severe |
| Follow-up1-HRSD-Diurnal Variation | 0=no variation, 1=worse in A.M., 2=worse in P.M. |
| Follow-up1-HRSD-Depersonalization and Derealization | 0=absent, 1=mild, 2=moderate, 3=severe, 4=incapacitating |
| Follow-up1-HRSD-Paranoid Symptoms | 0=none, 1=suspicious, 2=ideas of reference, 3=delusions of reference and persecution |
| Follow-up1-HRSD-Obsessional and compulsive Symptoms | 0=absent, 1=mild, 2=severe |
| Did patient commit suicide during study? | Patient committed suicide; 1=yes, 0=no, 99=unknown |
| suicide date | How many weeks after baseline |

*Note: These data were collected for each available follow-up time

**Supplemental Table S3**: Top SNP association regions for observed and imputed SNPs in the ISPC sample, along with replication analysis using AMPS and STAR*D data (replication results are based on the QIDS).

|  |  |  |  |  |  |  |  |  |  |  | ISPC | | AMPS | | STAR*D | |
| --- | --- | --- | --- | --- | --- | --- | --- | --- | --- | --- | --- | --- | --- | --- | --- | --- |
| Outcome | SNP | CHR | BP | I/O | gene | MA | CA | MAF | C-Frq | A-Frq | beta/ OR | P | beta/ OR | P | beta/ OR | P |
| %change in depression score | rs56058016 | 1 | 20609464 | I | VWA5B1 | T | C | 0.01 | 0.04 | 0.00 | -1.47 | 1.13E-07 | NA | NA | NA | NA |
|  | rs4747621 | 10 | 28770717 | I | LOC220906/ WAC | C | A | 0.32 | 0.38 | 0.28 | -0.28 | 1.75E-07 | -0.07 | 0.31 | 0.02 | 0.66 |
|  | rs7041589 | 9 | 110965171 | I | LOC100128657 | G | C | 0.07 | 0.18 | 0.01 | -0.49 | 5.40E-07 | -0.03 | 0.71 | -0.05 | 0.25 |
|  | rs113243734 | 7 | 138697944 | I | ZC3HAV1L | C | T | 0.01 | 0.03 | 0.00 | 1.13 | 5.64E-07 | -0.15 | 0.42 | -0.03 | 0.74 |
|  | rs9328202 | 6 | 3887339 | O | RPS25P7 | A | C | 0.03 | 0.04 | 0.02 | 0.72 | 6.15E-07 | 0.28 | 0.16 | NA | NA |
|  | rs11989215 | 8 | 6395909 | O | ANGPT2/ MCPH1 | G | A | 0.36 | 0.36 | 0.36 | 0.24 | 7.46E-07 | -0.02 | 0.78 | 0.00 | 0.98 |
|  | rs16855294 | 2 | 169199695 | O | STK39 | A | C | 0.42 | 0.6 | 0.32 | -0.24 | 8.82E-07 | 0.03 | 0.63 | -0.03 | 0.44 |
|  | rs12355629 | 10 | 68504487 | I | CTNNA3 | C | T | 0.02 | 0.05 | 0.00 | -1.02 | 1.04E-06 | -0.19 | 0.41 | -0.13 | 0.34 |
|  | rs9520830 | 13 | 108935308 | I | TNFSF13B | C | A | 0.04 | 0.08 | 0.02 | 1.06 | 1.24E-06 | 0.34 | 0.07 | NA | NA |
|  | rs73198127 | 8 | 17442885 | I | PDGFRL | A | G | 0.09 | 0.25 | 0.01 | -0.41 | 1.68E-06 | -0.03 | 0.72 | -0.08 | 0.07 |
| response | rs3782401 | 12 | 54911983 | I | NCKAP1L | A | G | 0.42 | 0.17 | 0.55 | 2.13 | 7.03E-07 | 1.02 | 0.90 | 0.96 | 0.80 |
|  | rs1348903 | 2 | 167386717 | I | SCN7A | T | C | 0.45 | 0.62 | 0.36 | 1.67 | 2.85E-06 | 0.85 | 0.25 | 1.00 | 0.97 |
|  | rs4747621 | 10 | 28770717 | I | LOC220906/ WAC | C | A | 0.32 | 0.38 | 0.28 | 1.72 | 2.88E-06 | 1.05 | 0.73 | 0.95 | 0.56 |
|  | rs17236056 | 15 | 33720854 | I | RYR3 | A | G | 0.12 | 0.18 | 0.09 | 0.35 | 3.52E-06 | 1.21 | 0.27 | 1.17 | 0.13 |
|  | rs41273882 | 6 | 53517228 | I | KLHL31 | C | A | 0.08 | 0.08 | 0.08 | 0.41 | 5.06E-06 | 0.91 | 0.71 | 1.13 | 0.39 |
|  | rs8088956 | 18 | 4973174 | I | PPIAP14 | A | G | 0.47 | 0.52 | 0.44 | 1.59 | 6.19E-06 | 0.91 | 0.53 | 0.95 | 0.56 |
|  | rs11989215 | 8 | 6395909 | O | ANGPT2/ MCPH1 | G | A | 0.36 | 0.36 | 0.36 | 0.62 | 6.70E-06 | 1.18 | 0.27 | 0.97 | 0.74 |
|  | rs114548043 | 6 | 32963844 | I | HLA-DOA | A | T | 0.05 | 0.02 | 0.07 | 0.27 | 1.00E-05 | 0.68 | 0.36 | NA | NA |
|  | rs12024635 | 1 | 65568853 | I | MRPS21P1 | A | T | 0.14 | 0.08 | 0.16 | 0.51 | 1.23E-05 | 0.96 | 0.90 | 0.94 | 0.63 |
|  | rs11120788 | 1 | 6992858 | I | CAMTA1 | A | G | 0.24 | 0.34 | 0.19 | 1.71 | 1.24E-05 | 0.91 | 0.51 | 0.98 | 0.81 |

I/O = indicator of whether the SNP was observed (O) or imputed (I) in ISPC; CA = common allele; MA = minor allele (in ISPC); MAF = minor allele frequency; C-Frq = Frequency of MA in subset; A-Frq = Frequency of MA in Asian subset; Beta/OR = regression parameter estimates (beta) for “MA” effect on quantitative trait outcome or odds ratio (OR) estimate for SNP effect on binary outcome

**Supplemental Table S4:** Replication of top results from recent large-sample pharmacogenomics GWAS of antidepressant response^23^^,^ ^24^

| SNP | gene | chr | Prior evidence | | ISPC %change | | ISPC response | | Meta-analysis* %change | | Meta-analysis* response | |
| --- | --- | --- | --- | --- | --- | --- | --- | --- | --- | --- | --- | --- |
|  |  |  | Associated Outcome | P | beta | P | OR | P | beta | P | OR | P |
| rs17651119 | MYO10 | 5 | 12 week %improvement, entire sample ^24^ | 1.78E-08 | 0.060 | 0.39 | 0.89 | 0.46 | -0.06 | 0.44 | 0.91 | 0.53 |
| rs2546057 | KCTD15 | 19 | 12 week %improvement, entire sample ^24^ | 2.56E-07 | -0.110 | 0.076 | 1.33 | **0.027** | -0.03 | 0.53 | 1.06 | 0.53 |
| rs12410462 | -- | 1 | 12 week %improvement, entire sample ^24^ | 4.20E-07 | 0.040 | 0.60 | 0.93 | 0.64 | 0.00 | 0.93 | 1.01 | 0.93 |
| rs7174755 | ITGA11 | 15 | 2 week % improvement, entire sample ^24^ | 9.12E-07 | 0.090 | 0.24 | 0.89 | 0.47 | -0.13 | **0.010** | 0.79 | **0.025** |
| rs10065906 |  | 5 | 2 week partial response, entire sample ^24^ | 5.29E-08 | 0.020 | 0.74 | 0.96 | 0.71 | -0.07 | 0.079 | 0.87 | 0.12 |
| rs17538444 | ENOX1 | 13 | 12 week %improvement, SSRI subset ^24^ | 4.17E-07 | -0.160 | 0.33 | 1.33 | 0.42 | 0.20 | 0.066 | 1.52 | 0.071 |
| rs12054895 | -- | 5 | 2 week % improvement, SSRI subset ^24^ | 2.65E-08 | -0.040 | 0.44 | 1.11 | 0.33 | 0.018 | 0.67 | 0.93 | 0.41 |
| rs4585146 | FGF12 | 3 | 2 week % improvement, SSRI subset ^24^ | 3.00E-07 | 0.030 | 0.59 | 0.99 | 0.91 | 0.02 | 0.59 | 0.97 | 0.73 |
| rs10818702 | ORIJ2 | 9 | Response to any antidepressant ^23^ | 2.19E-06 | -0.070 | 0.21 | 0.99 | 0.95 | -0.01 | 0.67 | 0.97 | 0.71 |
| rs11624702 | MDGA2 | 14 | Response to any antidepressant ^23^ | 4.08E-06 | 0.040 | 0.37 | 0.83 | **0.061** | -0.002 | 0.95 | 0.95 | 0.36 |
| rs7708972 | ADAMTS6 | 5 | Response to serotonergic antidepressant ^23^ | 2.49E-06 | -0.010 | 0.91 | 0.93 | 0.47 | -0.01 | 0.69 | 0.98 | 0.67 |
| rs1493451 | ADAMTS6 | 5 | Response to serotonergic antidepressant ^23^ | 2.35E-06 | -0.040 | 0.37 | 1.05 | 0.64 | -0.01 | 0.65 | 1.00 | 0.97 |
| rs10515893 | -- | 5 | Response to serotonergic antidepressant ^23^ | 1.37E-06 | 0.050 | 0.45 | 0.86 | 0.26 | 0.01 | 0.86 | 0.92 | 0.25 |
| rs10783282 | ADCY6 | 12 | Response to serotonergic antidepressant ^23^ | 1.16E-06 | 0.020 | 0.66 | 0.92 | 0.49 | 0.01 | 0.80 | 1.05 | 0.40 |

*Note that for SNPs previously reported by Tansey et al ^23^, we tested replication using our ISPC sample, and our combined ISPC+AMPS+STAR*D meta-analysis. However, for SNPs previously reported by Uher et al. ^24^, we tested for replication using our ISPC sample, and our combined ISPC+AMPS analysis (without STAR*D) because the analysis of Uher et al. included STAR*D samples.
**Supplemental Figure S1**: Structure analysis plot for ISPC subjects.

**Supplemental Figure S2**: QQ plots for primary analyses of ISPC data including observed and imputed SNPs (A) %ΔHRSD (B) Response

B

A

**Supplemental Figure S3**: QQ plots for meta-analyses of ISPC, AMPS and STAR*D data (A) %Δ depression score (B) Response

A

B

**Supplemental Figure S4**: LocusZoom plot of results from meta-analysis of “response” in the *NRG1* region

Top 5 SNPs in Figure S5

| SNP | CHR | BP | I/O* | OR | pvalue | gene |
| --- | --- | --- | --- | --- | --- | --- |
| rs10954808 | 8 | 31479623 | I | 0.73 | 1.20E-06 | NRG1 |
| rs12680816 | 8 | 31477431 | I | 0.73 | 1.39E-06 | NRG1 |
| rs12546600 | 8 | 31488328 | I | 0.73 | 1.56E-06 | NRG1 |
| rs12546840 | 8 | 31490319 | I | 0.72 | 3.09E-06 | NRG1 |
| rs12541452 | 8 | 31488275 | IO | 1.36 | 3.15E-06 | NRG1 |

*I/O indicator of whether the SNP was observed (O) or imputed (I). IO means the SNP was observed in at least one of the datasets contributing to the meta-analysis and imputed in at least one dataset.
